# The Gut–Immune–Brain Axis in Aging: Integrating Immunosenescence, Inflammaging, and Neuroinflammation for Precision Medicine

**DOI:** 10.3390/medsci14050536

**Published:** 2026-08-31

**Authors:** Dejana Bajić, Jelena Vučković, Nikolina Pupovac, Danijel Slavić, Mirjana Stojšić, Nikola Hodoba, Nemanja Todorović, Milica Plazačić, Nataša Milošević

**Affiliations:** 1 Department of Biochemistry, Faculty of Medicine, University of Novi Sad, 21000 Novi Sad, Serbia; 015349@mf.uns.ac.rs; 2 Department of Anesthesiology and Perioperative Medicine, Faculty of Medicine, University of Novi Sad, 21000 Novi Sad, Serbia; jelena.vuckovic@mf.uns.ac.rs; 3 Institute for Cardiovascular Diseases of Vojvodina, Put Doktora Goldmana 4, 21204 Sremska Kamenica, Serbia; 4 Department of Anatomy, Faculty of Medicine, University of Novi Sad, 21000 Novi Sad, Serbia; nikolina.pupovac@mf.uns.ac.rs; 5 Department of Physiology, Faculty of Medicine, University of Novi Sad, 21000 Novi Sad, Serbia; danijel.slavic@mf.uns.ac.rs; 6 Department of Pediatrics, Faculty of Medicine, University of Novi Sad, 21000 Novi Sad, Serbia; mirjana.stojsic@mf.uns.ac.rs (M.S.); milica.plazacic@mf.uns.ac.rs (M.P.); 7 Institute for Child and Youth Health Care of Vojvodina, 21000 Novi Sad, Serbia; 8 Department of Pharmacy, Faculty of Medicine, University of Novi Sad, 21000 Novi Sad, Serbia; nemanja.todorovic@mf.uns.ac.rs (N.T.); natasa.milosevic@mf.uns.ac.rs (N.M.)

**Keywords:** gut microbiota, aging, neuroinflammation, immunosenescence, inflammaging, microglia, blood–brain barrier, short-chain fatty acids, precision medicine, Alzheimer’s disease

## Abstract

**Background:** Population aging is accompanied by progressive immune remodeling, chronic low-grade inflammation, and increased susceptibility to neurodegenerative diseases. Although the microbiota–gut–brain axis is increasingly recognized as a regulator of neuroimmune homeostasis, mechanisms linking age-associated dysbiosis with immunosenescence, barrier dysfunction, and brain aging remain incompletely understood. This review integrates current evidence into the proposed Gut–Immune–Brain Resilience Axis (GIBRA), a systems-level model describing how microbial signaling shapes neuroimmune resilience during aging. **Methods:** A structured narrative review was conducted using PubMed and the Web of Science Core Collection from database inception up to July 2026. Evidence from systematic reviews, meta-analyses, consensus statements, mechanistic and translational studies, longitudinal cohorts, randomized clinical trials, and observational studies was synthesized. **Results:** Current evidence supports an important role for the disruption of microbial functional signaling in neuroimmune aging, while microbial taxonomy and functional profiles provide complementary levels of biological information. Reduced short-chain fatty acid production, dysregulated tryptophan metabolism, microbial translocation, endotoxin-mediated innate immune activation, and gut-conditioned adaptive immune responses promote immunosenescence, inflammaging, barrier dysfunction, and microglial priming. The proposed Double-Barrier Hypothesis links intestinal and blood–brain barrier dysfunction as complementary mechanisms underlying chronic neuroinflammation. GIBRA highlights functional microbiome endotypes, biomarkers, multi-omics, and artificial intelligence as emerging tools for precision medicine. **Conclusions:** GIBRA provides an integrated systems biology perspective connecting microbial signaling, immune resilience, barrier integrity, and brain resilience during aging. Prioritizing functional resilience over microbial taxonomy may improve biomarker discovery, patient stratification, and microbiome-targeted interventions for neurodegenerative disease prevention. Prospective longitudinal studies integrating multi-omics are needed to support clinical translation.

## 1. Introduction

Population aging is one of the defining demographic challenges of the twenty-first century and is expected to substantially increase the global burden of chronic age-related diseases. According to World Health Organization projections, the global population aged 60 years and older is expected to reach approximately 2.1 billion by 2050 [1]. Disorders affecting the nervous system already constitute the leading global cause of health loss, with neurodegenerative diseases representing a rapidly expanding component of this burden [2]. Alzheimer’s disease accounts for approximately 60–70% of dementia cases worldwide [3], while Parkinson’s disease has shown one of the most rapidly increasing burdens among neurological disorders [4,5]. Despite major advances in molecular neuroscience, therapeutic options remain limited: most treatments are symptomatic, although anti-amyloid monoclonal antibodies have demonstrated modest disease-modifying effects in selected patients with early Alzheimer’s disease, whereas no established disease-modifying therapy is currently available for Parkinson’s disease [6,7]. These limitations underscore the urgent need to identify upstream mechanisms that drive neurodegeneration and may provide novel opportunities for disease prevention and intervention.

Traditionally, neurodegenerative disorders have been interpreted primarily through the lens of neuronal dysfunction, protein aggregation, and genetic susceptibility. However, growing evidence indicates that aging is accompanied by profound remodeling of both innate and adaptive immunity that contributes to the initiation and progression of age-related diseases [8,9]. Consequently, immunosenescence and inflammaging have emerged as two interrelated manifestations of biological aging [8,9,10]. Immunosenescence is characterized by reduced adaptive immune diversity, impaired immune surveillance, altered innate immune function, and the accumulation of senescent or terminally differentiated immune-cell populations, whereas inflammaging reflects persistent, low-grade, largely sterile inflammation involving mediators such as IL-6, TNF-α, and IL-1β [9,10,11]. Together, these processes promote tissue dysfunction and increase the susceptibility to frailty, metabolic and cardiovascular disorders, and neurodegeneration [9,11].

The central nervous system is particularly vulnerable to age-associated immune dysregulation. Chronic neuroinflammation is increasingly recognized as a major feature of brain aging and an important contributor to neurodegenerative disease progression [12,13]. Among the cellular mediators involved, microglia—the resident macrophage population of the central nervous system—play a central role in immune surveillance, synaptic maintenance, tissue repair, and neuroimmune homeostasis [12,14]. During aging, microglia undergo transcriptional, metabolic, morphological, and functional reprogramming and may acquire primed or senescence-associated phenotypes characterized by exaggerated inflammatory responses, impaired resolution and repair, and increased reactivity to secondary insults [13,14,15]. Consequently, age-associated microglial dysfunction and priming are regarded as important mechanistic links between systemic immune aging and progressive neurodegeneration [13,14,15].

Increasing attention has focused on the gut microbiome as a potential upstream regulator of neuroimmune aging. Beyond their established roles in digestion and metabolism, intestinal microorganisms generate a diverse repertoire of bioactive metabolites and signaling structures—including short-chain fatty acids, tryptophan-derived metabolites, secondary bile acids, and microbial extracellular vesicles—that regulate immune function, epithelial barrier integrity, metabolism, and host homeostasis [16,17,18]. These microbial signals extend beyond the gastrointestinal tract and participate in systemic communication networks that influence distant organs, including the brain [16,17].

These observations have led to recognition of the microbiota–gut–brain axis as a dynamic, bidirectional communication network integrating the intestinal microbiota, peripheral immune system, enteric, and central nervous systems through metabolic, neural, endocrine, and immunological pathways [16,19]. Aging is associated with substantial, although heterogeneous, alterations in microbial composition and function, including changes in diversity, depletion of selected beneficial metabolite-producing taxa, expansion of opportunistic organisms, altered metabolite production, and impaired intestinal barrier integrity [8,20,21,22]. Together, these changes may promote microbial translocation, systemic inflammation, and chronic immune activation. Accordingly, age-associated dysbiosis may function not merely as a biomarker of aging, but in susceptible individuals, as an active contributor to neuroimmune dysfunction [20,21,22]. These observations further suggest that neuroimmune aging may be understood as progressive loss of resilience across an interconnected gut–immune–brain network rather than as dysfunction of isolated organs.

Despite substantial progress in characterizing individual components of the microbiota–gut–brain axis, an integrative understanding of how age-associated microbial alterations converge with immune remodeling, barrier dysfunction, and microglial activation to shape neuroimmune resilience across the aging trajectory remains incomplete [16,20,23]. Although numerous studies have reported associations between gut microbiome composition and neurodegenerative diseases, causal direction and the mechanisms connecting age-related dysbiosis with neurodegeneration remain insufficiently resolved [16,23]. Emerging evidence suggests that the disruption of microbial functional capacity and signaling—rather than the loss of individual microbial species alone—may be particularly relevant to neuroimmune aging [17,20,23]. Through coordinated alterations in microbial metabolites, microbial-associated molecular patterns, immune mediators, and barrier integrity, age-associated dysbiosis may amplify systemic inflammation, promote immune remodeling, facilitate microglial priming, and ultimately increase vulnerability to neurodegenerative disease [16,23,24].

**Figure 1:**
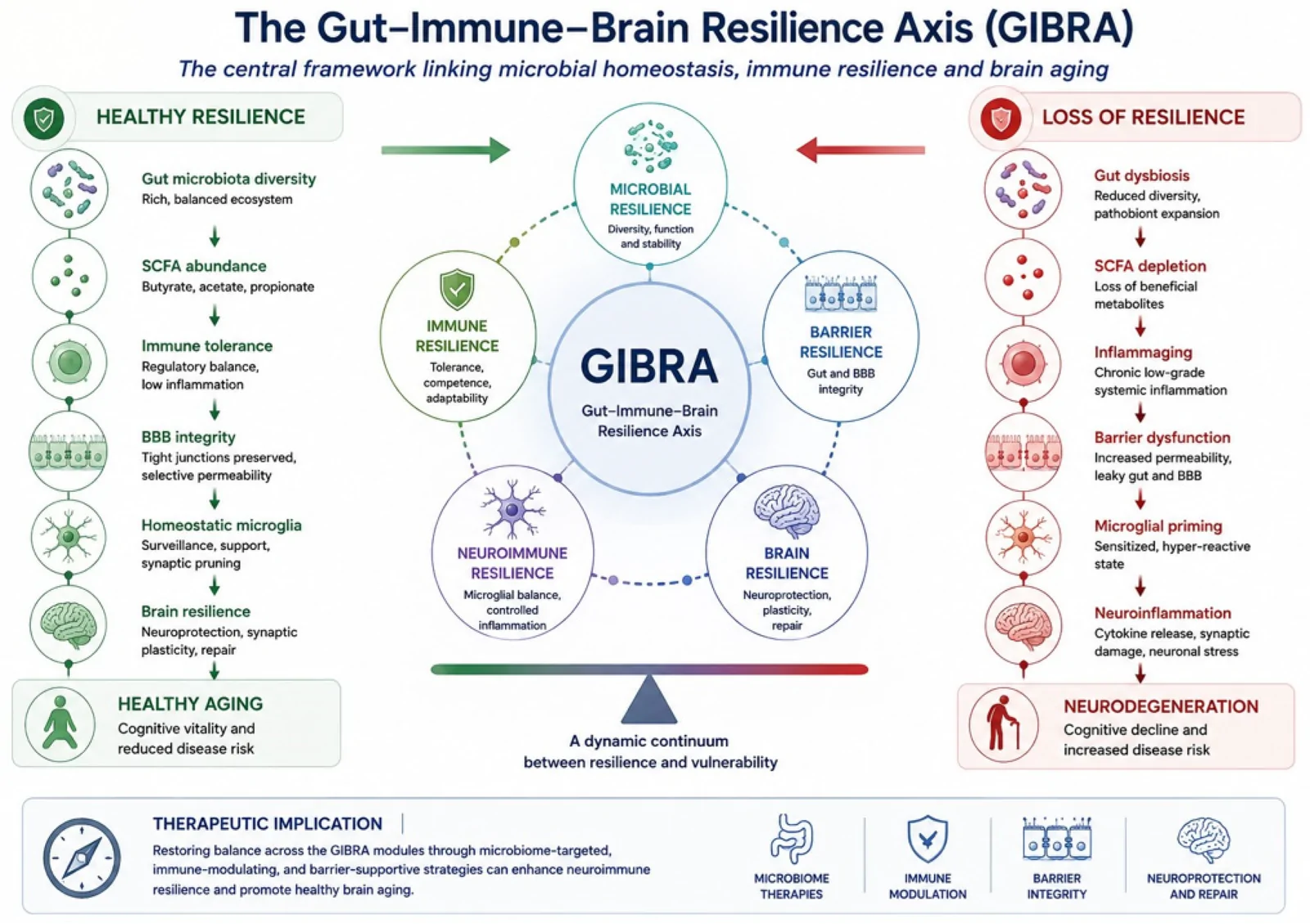
The Gut–Immune–Brain Resilience Axis (GIBRA): a conceptual framework linking microbial homeostasis, immune resilience, barrier integrity, and brain resilience during aging. GIBRA conceptualizes healthy brain aging as a dynamic property emerging from coordinated interactions among the gut microbiota, immune system, epithelial and blood–brain barriers, and the central nervous system. The left panel illustrates biological processes that preserve resilience, including microbial homeostasis, beneficial metabolite production, immune tolerance, barrier integrity, and homeostatic microglial function. The right panel depicts the progressive loss of resilience driven by age-associated dysbiosis, depletion of microbial metabolites, chronic low-grade inflammation, barrier dysfunction, microglial priming, and neuroinflammation, ultimately increasing susceptibility to neurodegeneration. The central modules emphasize microbial, immune, barrier, neuroimmune, and brain resilience as interconnected components of a dynamic systems network, while the lower panel highlights resilience as a continuum between healthy aging and disease and outlines potential microbiome-targeted therapeutic strategies.

To integrate these interconnected biological processes into a unified conceptual framework, we propose the Gut–Immune–Brain Resilience Axis (GIBRA) (Figure 1). Rather than viewing the gut microbiota, immune system, epithelial and blood–brain barriers, and central nervous system as independent biological entities, GIBRA conceptualizes these systems as a dynamic resilience network that collectively governs neuroimmune homeostasis throughout aging. Unlike conventional microbiota–gut–brain axis models, which primarily describe bidirectional communication pathways between the gut and the brain, GIBRA reframes these interactions through the systems-level concept of resilience across aging. Its conceptual extension lies in linking microbial functional signaling, immune competence, intestinal and blood–brain barrier integrity, and neuroimmune regulation to individual trajectories of resilience and vulnerability to neurodegeneration. Within this framework, microbial homeostasis, preserved microbial signaling capacity, immune competence, barrier integrity, and tightly regulated microglial activity represent complementary determinants of neuroimmune resilience. Conversely, progressive dysbiosis, impaired microbial signaling, chronic low-grade inflammation, barrier dysfunction, and microglial priming are viewed as converging mechanisms that progressively erode resilience and increase susceptibility to cognitive decline and neurodegeneration. Importantly, GIBRA shifts the focus from individual pathogenic pathways toward resilience as an integrative systems-level property that may help explain interindividual differences in healthy brain aging and disease susceptibility. By integrating these biological processes into a unified systems perspective, the GIBRA framework may facilitate hypothesis generation, biomarker discovery, and the development of resilience-oriented preventive and therapeutic strategies for healthy brain aging.

The aim of this review is to synthesize current evidence supporting the hypothesis that age-associated dysbiosis may act as an upstream contributor to neuroimmune aging through the disruption of microbial signaling networks. We examine how age-related alterations in the gut microbiome may contribute to immunosenescence, inflammaging, barrier dysfunction, and microglial priming, and discuss the molecular mechanisms linking these processes to neurodegenerative vulnerability. Finally, we consider how emerging multi-omics technologies and microbiome-targeted interventions may support a transition from descriptive associations toward mechanism-based, precision approaches aimed at preserving neuroimmune resilience during aging.

### Literature Search and Scope of the Review

This review was designed as a structured narrative review to synthesize current evidence on the role of the gut microbiome in neuroimmune aging and to integrate mechanistic, translational, and clinical perspectives within the proposed Gut–Immune–Brain Resilience Axis (GIBRA) framework. Because the objective was to integrate multiple interconnected biological processes—including immunosenescence, inflammaging, microbial signaling, barrier dysfunction, microglial activation, biomarker development, and precision microbiome medicine—rather than to address a single focused clinical question, a structured narrative approach was considered the most appropriate.

The literature search was performed using PubMed and the Web of Science Core Collection from database inception through July 2026. Search terms included combinations of gut microbiome, gut–brain axis, gut–immune–brain axis, aging, healthy aging, immunosenescence, inflammaging, microglia, microglial priming, neuroinflammation, intestinal barrier, blood–brain barrier, gut permeability, microbial metabolites, short-chain fatty acids, SCFAs, indoles, tryptophan metabolism, secondary bile acids, extracellular vesicles, postbiotics, microbial signaling, multi-omics, metabolomics, biomarkers, artificial intelligence, precision medicine, Alzheimer’s disease, and Parkinson’s disease. Additional relevant publications were identified through the manual screening of reference lists from key articles, landmark reviews, consensus statements, and recent high-impact publications.

Priority was given to systematic reviews, meta-analyses, consensus statements, mechanistic studies, translational research, longitudinal human cohorts, randomized clinical trials, and clinically relevant observational studies published in peer-reviewed journals. Historical publications were included selectively when necessary to provide scientific context or illustrate the evolution of concepts discussed in this review. Particular emphasis was placed on studies investigating functional microbial signaling, host–microbiome interactions, biomarkers of neuroimmune aging, and emerging precision medicine approaches, reflecting the conceptual focus of the proposed GIBRA framework.

For inclusion in the narrative synthesis, priority was given to studies directly addressing one or more of the principal domains of the review—microbial composition or function, microbial signaling, immune aging, intestinal or neurovascular barrier integrity, neuroinflammation, cognitive or neurodegenerative phenotypes, and translational interventions—with preference for human and longitudinal evidence where available. Studies were interpreted according to their design and evidentiary level, with observational human studies used primarily to establish clinical associations and temporal relationships, and experimental studies used to assess mechanistic plausibility rather than direct clinical causality. Because this was a structured narrative review rather than a systematic review, no preregistered protocol, formal PRISMA-based study-selection procedure, or study-level risk-of-bias assessment was performed; residual selection bias and subjectivity in evidence selection therefore cannot be completely excluded.

## 2. The Aging Microbiome: From Homeostasis to a Senescent Phenotype

### 2.1. Characteristics of a Healthy Adult Microbiome

The adult human gut microbiome represents a highly complex microbial ecosystem comprising trillions of microorganisms that collectively contribute to nutrient metabolism, immune regulation, epithelial integrity, and systemic homeostasis [16,25]. Although considerable interindividual variability exists and no universal taxonomic definition of a “healthy microbiome” has been established, healthy gut microbial ecosystems are generally characterized by ecological stability, functional diversity and redundancy, and resilience to environmental perturbations [16,26,27]. These properties enable microbial communities to preserve essential biological functions despite fluctuations in the abundance or loss of individual taxa [26,27].

**Figure 2:**
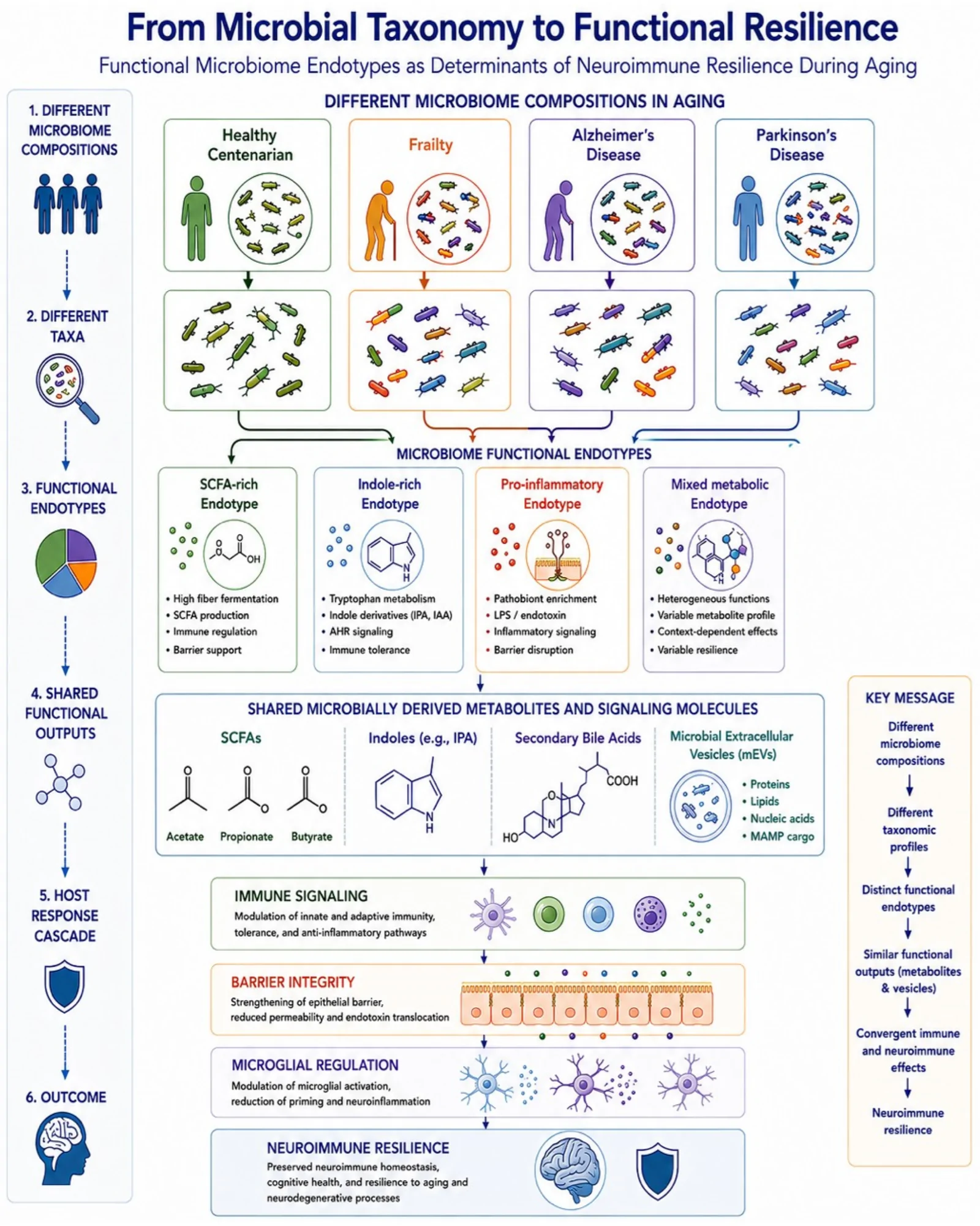
Functional microbiome endotypes as potential determinants of neuroimmune resilience during aging. Microbial communities observed in healthy centenarians, frail older adults, and patients with Alzheimer’s or Parkinson’s disease may differ substantially in taxonomic composition. Nevertheless, the proposed model suggests that neuroimmune resilience may depend not only on taxonomic composition, but also on the preservation of key microbial functions. Production of bioactive metabolites—including short-chain fatty acids (SCFAs), tryptophan-derived indoles, and secondary bile acids—as well as vesicle-mediated delivery of microbial proteins, lipids, nucleic acids, and immunologically active molecular cargo by microbial extracellular vesicles (mEVs), represent complementary dimensions of microbial functional output. These signals may influence immune homeostasis, epithelial and blood–brain barrier integrity, and microglial regulation. Accordingly, functional microbiome endotypes may be defined not only by microbial composition or metabolite abundance, but by the integrated capacity of microbial communities to generate metabolic and vesicle-mediated signals relevant to host neuroimmune resilience. Conversely, disruption of these functional outputs may contribute to immune dysregulation, barrier dysfunction, neuroinflammation, and progressive loss of neuroimmune resilience. The figure illustrates the hypothesis that functional microbiome endotypes may provide a more mechanistically informative framework for understanding healthy brain aging than taxonomic composition alone.

Importantly, the contribution of the microbiome to host health is not determined solely by its taxonomic composition but also by its functional capacity, because taxonomically distinct microbial communities may retain overlapping metabolic functions through functional redundancy [26] (Figure 2). Functionally diverse microbial communities contribute to nutrient metabolism, vitamin synthesis, bile acid transformation, maintenance of epithelial integrity, and the regulation of innate and adaptive immunity [16,28,29]. A defining feature of a functionally competent microbiome is therefore its capacity to generate a broad repertoire of bioactive metabolites that participate in host–microbe communication networks [16,28,29]. Human studies of aging and longevity indicate substantial heterogeneity in both microbiome composition and functional potential, with centenarians and successfully aging older adults frequently displaying microbial features distinct from those observed in frailty and age-related disease [30].

Among these metabolites, the short-chain fatty acids (SCFAs) acetate, propionate, and butyrate are major products of the microbial fermentation of dietary fiber and important mediators of host–microbiome communication [31]. SCFAs—particularly butyrate, which serves as a major energy substrate for colonocytes—also function as signaling molecules that regulate epithelial barrier integrity, immune-cell differentiation, inflammatory responses, and communication with extraintestinal organs, including the brain [31]. These effects are mediated, at least in part, through the activation of G-protein-coupled receptors and epigenetic regulation involving the inhibition of histone deacetylases [31,32]. Collectively, SCFA signaling is increasingly recognized as an important contributor to host homeostasis and may support healthy aging by promoting epithelial integrity, immune regulation, and metabolic resilience [30,31,32].

Representative quantitative human evidence supporting these phenotype-associated functional profiles is summarized in Table 1; however, because available studies differ substantially in cohort design, biological matrices, analytical platforms, and covariate adjustment, these data should not be interpreted as direct cross-phenotype statistical comparisons.

### 2.2. Age-Associated Dysbiosis: Loss of Diversity and Functional Capacity

Aging is associated with substantial but heterogeneous alterations in gut microbial composition and function, shaped not only by chronological age but also by frailty, diet, medication use, comorbidities, lifestyle, and living environment [42,43,44]. Although no universal “aging microbiome signature” has been identified, unhealthy aging and frailty are frequently accompanied by reduced microbial diversity and ecological stability, depletion of beneficial commensals, and the enrichment of opportunistic or pro-inflammatory taxa [42,43,44,45].

A recurrent feature of unhealthy aging is the reduced abundance of short-chain fatty acid-producing bacteria, including members of the genera *Faecalibacterium*, *Roseburia*, and *Eubacterium*, although the consistency of individual taxonomic associations varies across cohorts [43,44,45,46]. Among these organisms, *Faecalibacterium prausnitzii* is a major butyrate producer with well-described immunoregulatory and anti-inflammatory properties [46,47]. Lower abundance of *F. prausnitzii* has been associated with frailty and adverse metabolic and inflammatory profiles, while reviews of older populations also link the depletion of SCFA-producing bacteria with cognitive decline and reduced fecal butyrate concentrations [44,46,48].

In parallel, unhealthy aging may be accompanied by the enrichment of pathobionts—commensal organisms that can promote disease under conditions of disrupted ecological or host homeostasis—and by the increased representation of taxa belonging to the phylum Proteobacteria, including members of the family Enterobacteriaceae [42,43,49]. Such compositional changes may increase the immunogenic potential of the intestinal microbiota, and together with impaired epithelial barrier integrity, promote microbial-product translocation, endotoxin-associated signaling, and chronic activation of innate inflammatory pathways [43,49,50].

Importantly, age-associated dysbiosis should not be interpreted solely as a shift in bacterial taxonomy. Rather, it may involve the remodeling of microbial functional capacity, including the reduced production of protective metabolites and altered regulation of epithelial, metabolic, and immune homeostasis [21,42,43]. From a mechanistic perspective, impaired microbial signaling may be more biologically relevant than the loss of any single bacterial species. Reductions in SCFA-producing capacity, alterations in tryptophan and bile-acid metabolism, increased microbial translocation, and enhanced pro-inflammatory signaling may collectively promote systemic inflammation and influence microglial homeostasis [21,43,51,52]. Importantly, however, these functional and metabolic alterations should not be interpreted as age-specific signatures, because their expression is strongly modified by dietary, pharmacological, lifestyle, environmental, and host-related factors, as discussed below.

#### 2.2.1. Interindividual Heterogeneity of Microbiome Aging

Although population-level studies have identified age-associated alterations in gut microbial composition and function, accumulating evidence indicates that there is no single “aging microbiome” [20,21,42,53]. Instead, older individuals exhibit substantial interindividual variability shaped by genetics, diet, medication use, comorbidities, geography, and lifestyle [20,42,53]. Healthy centenarians, frail older adults, and patients with Alzheimer’s or Parkinson’s disease frequently display distinct microbial configurations rather than a common dysbiotic profile. These observations suggest that neuroimmune aging is more accurately characterized by multiple microbiome endotypes with different functional and immunological consequences rather than by a universal age-associated microbial signature [26,53]. Recognizing this heterogeneity has important translational implications because distinct microbiome endotypes may differ not only in microbial composition, but also in their functional capacity to regulate immune homeostasis, barrier integrity, and neuroimmune resilience. These observations further suggest that functional microbial characteristics may provide biologically relevant information complementary to taxonomic composition when characterizing heterogeneous trajectories of healthy and unhealthy aging [26,30,53].

#### 2.2.2. Functional Resilience Beyond Microbial Taxonomy

Increasing evidence indicates that taxonomic composition alone may provide an incomplete representation of microbiome–host interactions because taxonomically distinct microbial communities can retain overlapping metabolic capabilities through functional redundancy [26,54,55]. Conversely, communities with broadly similar taxonomic profiles may differ substantially in gene content, transcriptional activity, metabolic flux, and metabolite production. Accordingly, functional profiling should not be viewed as a replacement for taxonomy, but as a complementary layer that may, in selected biological contexts, provide information more proximal to host phenotype.

Recent human multi-omics studies support this interpretation. In older adults, integrated gut metagenomic and circulating metabolomic profiling has identified microbial and metabolic signatures associated with frailty severity, adverse clinical phenotypes, and mortality risk, demonstrating that the integration of microbial and metabolic information can capture clinically relevant variation associated with aging-related phenotypes [46]. Similarly, studies of healthy aging and longevity have demonstrated that substantial taxonomic heterogeneity can coexist with the preservation or expansion of specific metabolic capacities, supporting the concept that microbial functional potential may remain conserved despite variation in community composition [53,54,56,57].

Complementary mechanistic evidence from integrated experimental multi-omics studies further supports the biological relevance of functional host–microbiome interactions. Using metagenomic, transcriptomic, metabolomic, and metabolic-modeling approaches, Best et al. demonstrated age-associated alterations in microbial metabolic activity and host–microbiome metabolic exchange involving pathways relevant to butyrate production, bile-acid metabolism, and host inflammatory and homeostatic responses across intestinal and extra-intestinal host tissues [58]. These findings further illustrate that age-related biological effects of the microbiome may not be fully captured by species-level abundance alone. However, because these observations were derived from experimental models, their translational relevance requires confirmation in longitudinal human cohorts.

These observations are consistent with the concept of functional redundancy, whereby multiple taxonomically distinct organisms can contribute to common metabolic pathways [26,55]. From the perspective of neuroimmune aging, therefore, the biological relevance of a microbial community may depend not only on which organisms are present, but also on whether the ecosystem preserves the capacity to generate or transform metabolites involved in epithelial integrity, immune regulation, and neuroimmune communication, including short-chain fatty acids, tryptophan-derived metabolites, and secondary bile acids [28,31,52,59].

Beyond SCFAs and tryptophan-derived metabolites, secondary bile acids and microbial extracellular vesicles (mEVs) broaden the concept of functional microbial output beyond conventional metabolite abundance. The microbiota-dependent transformation of primary into secondary bile acids generates bioactive signaling molecules that can engage receptors such as FXR and TGR5/GPBAR1, thereby influencing the metabolic, immune, barrier, and neuroinflammatory pathways. Human studies support the translational relevance of this axis. In a large ADNI cohort, altered circulating primary-to-secondary bile-acid profiles, including increased deoxycholic acid relative to cholic acid, were associated with cognitive impairment and Alzheimer’s disease [60]. In Parkinson’s disease, targeted metabolomic profiling demonstrated higher plasma concentrations of the secondary bile acids deoxycholic acid and glycodeoxycholic acid, with glycodeoxycholic acid independently associated with PD status [61]. Experimental aging studies further support a mechanistic involvement of this pathway: age-associated disruption of brain bile-acid homeostasis was linked to gut microbial alterations, microglial inflammation, neuroinflammation, and behavioral impairment in mice [62].

Microbial extracellular vesicles represent a mechanistically distinct functional output. These nanoscale membrane-bound structures can package and transport bacterial proteins, lipids, nucleic acids, metabolites, and immunologically active components, thereby enabling microbial signals to interact with host cells independently of intact bacterial translocation [63]. Emerging experimental evidence provides direct support for their relevance to the gut–brain axis. In an Alzheimer’s disease mouse model, germ-free conditions reduced Aβ pathology, microglial inflammatory activation, and synaptic deficits, whereas the administration of commensal gut microbiota-derived bacterial extracellular vesicles (bEVs) reversed several of these effects, including microglial activation and Aβ plaque accumulation [64]. Complementary translational evidence showed increased circulating LPS-containing bEVs in patients with Alzheimer’s disease; experimentally, these vesicles were capable of crossing the blood–brain barrier and promoting microglial activation and excessive C1q–C3 complement-mediated synaptic pruning through a mechanism involving microglial Piezo1 [65]. Together, these findings suggest that functional microbiome endotyping may ultimately require the characterization not only of metabolite production, but also of vesicle-mediated microbial signaling capacity.

Importantly, current evidence does not establish the universal superiority of functional over taxonomic profiling. Rather, it supports an integrated approach in which taxonomy, microbial gene content, transcriptional activity, and metabolite output are considered complementary levels of biological information. Such multidimensional profiling may provide a more mechanistically informative basis for defining functional microbiome endotypes than taxonomic composition alone.

Importantly, age-associated differences in microbial metabolite profiles should not be interpreted as specific consequences of chronological aging. SCFA availability is jointly shaped by microbial functional capacity, dietary substrate availability, particularly fermentable fiber intake, intestinal transit, medication exposure, physical activity, comorbidities, and host absorptive and metabolic processes [31,42,54,66,67]. Similarly, tryptophan metabolism reflects dynamic host–microbiome co-metabolism across the kynurenine, serotonin, and microbial indole pathways and is influenced by dietary tryptophan availability, microbial enzymatic capacity, systemic inflammatory signaling, host enzymatic activity, medication exposure, and metabolic or renal function [52,68,69,70,71]. Thus, chronological age represents only one component of a broader network of determinants shaping microbial and host–microbiome metabolic outputs. These sources of heterogeneity are considered further in Section 2.3.

### 2.3. Environmental and Host Determinants of Microbiome Aging and Function

The substantial interindividual variability observed in microbiome aging reflects the combined influence of environmental exposures, lifestyle factors, medication use, comorbidities, and host physiology, which can affect not only the microbial composition, but also microbial functional capacity and metabolite production [20,21,42,53,65,67].

Diet represents one of the strongest modifiable determinants of both microbiome composition and metabolic function throughout life [42,54,66]. Dietary substrate availability directly influences microbial fermentation; consequently, reduced intake of fermentable fiber and plant-derived foods, together with a greater reliance on highly processed diets, may reduce the abundance and metabolic activity of SCFA-producing bacteria and alter SCFA availability [31,42,66]. In contrast, adherence to Mediterranean-style dietary patterns has been associated with the preservation of microbial diversity and enrichment of beneficial metabolite-producing taxa [42,66].

Physical activity represents another important modulator of microbial health [42,72]. Regular physical activity has been associated with greater microbial diversity, differences in SCFA-producing capacity, and more favorable metabolic profiles, whereas sedentary behavior has been linked to dysbiosis and systemic inflammation [31,72].

Frailty, a multidimensional syndrome characterized by reduced physiological reserve and increased vulnerability to stressors, is increasingly recognized as both a consequence and a driver of microbiome dysfunction [44,45,46,48]. Frail older adults often exhibit reduced microbial diversity, a lower abundance of beneficial commensals, and the enrichment of pro-inflammatory microbial communities. Because frailty prevalence increases with chronological age while independently influencing diet, physical activity, intestinal physiology, medication burden, and microbial ecology, it represents an important potential confounder in studies attributing microbiome alterations specifically to aging.

Polypharmacy constitutes an additional challenge in aging populations [20,42,67]. Older individuals are frequently exposed to multiple medications, many of which exert direct or indirect effects on the microbiome [67]. Proton pump inhibitors, nonsteroidal anti-inflammatory drugs, metformin, statins, and antidepressants have all been shown to alter microbial composition and metabolic activity. Among these exposures, antibiotic use remains particularly important due to its potential to induce long-lasting reductions in microbial diversity and resilience [67,73]. Importantly, medication exposure may influence not only taxonomic composition, but also microbial enzymatic activity and metabolite production, thereby confounding associations between chronological age and functional microbiome profiles [67].

Host-related physiological factors further contribute to this variability. Comorbidity burden, metabolic and renal function, intestinal transit, inflammatory status, and age-associated changes in host metabolism can modify both the intestinal environment and circulating metabolite concentrations [31,52,54,74]. These factors are particularly relevant when interpreting SCFA and tryptophan-related metabolite profiles because the measured intestinal or systemic concentrations reflect the combined effects of microbial production, host absorption, biotransformation, tissue utilization, and elimination rather than microbial activity alone [31,52,70,73]. Sex should also be considered as an additional source of biological heterogeneity within GIBRA, as sex-dependent differences may influence microbiome–metabolite–immune relationships and thereby modify individual neuroimmune resilience trajectories. Accordingly, sex should be incorporated as a relevant stratification variable in future longitudinal and biomarker-based studies.

Taken together, current evidence indicates that microbiome aging reflects the interaction of chronological age with environmental exposures, lifestyle patterns, medication use, comorbidities, and host physiology rather than an autonomous effect of aging alone [20,21,42,53,54,67]. These determinants can reshape both microbial ecology and functional output, thereby contributing to substantial interindividual variation in SCFA availability, tryptophan metabolism, immune signaling, and barrier homeostasis. Accordingly, age-associated dysbiosis should be viewed as a dynamic and functionally heterogeneous process whose biological consequences emerge from continuous host–microbiome–environment interactions [17,20,21,42,51,52,53,54]. This distinction has important translational implications because potentially modifiable determinants of microbial function may represent targets for preserving immune, barrier, and neuroimmune resilience across aging.

Future longitudinal and multi-omics studies should therefore systematically assess and report major potential confounders, including dietary intake, medication exposure, physical activity, frailty status, comorbidity burden, intestinal transit characteristics, and relevant host metabolic variables, and incorporate appropriate adjustment or stratification strategies when evaluating associations between chronological age, microbial function, metabolite profiles, and clinical phenotypes.

## 3. The Double-Barrier Hypothesis: Leaky Gut Meets Leaky Brain

Aging is increasingly recognized as a process characterized not only by cellular and molecular alterations, but also by the progressive deterioration of biological barriers that normally maintain compartmentalization between the host and the external environment [8,9,10,43]. Among these, the intestinal barrier and the blood–brain barrier (BBB) represent two of the most important interfaces protecting systemic and neural homeostasis. Emerging evidence suggests that dysfunction of these barriers is not an isolated phenomenon but rather a coordinated process linking age-associated dysbiosis, systemic inflammation, and neurodegeneration [23,24,75]. This concept, referred to here as the Double-Barrier Hypothesis, proposes that disruption of intestinal integrity promotes systemic inflammatory signaling, which subsequently compromises BBB function and facilitates neuroinflammation.

### 3.1. Intestinal Barrier Dysfunction

The intestinal barrier is a highly specialized multilayered structure designed to permit nutrient absorption while preventing the uncontrolled passage of microorganisms, toxins, and inflammatory molecules into the systemic circulation [75]. Its protective function depends on the coordinated interaction between the mucus layer, intestinal epithelial cells, immune components, and the resident microbiota [31,54,75].

The outer mucus layer serves as the first line of defense by physically separating luminal microorganisms from the intestinal epithelium [76]. This barrier is largely maintained through microbial–host interactions and is strongly influenced by microbial metabolites, particularly short-chain fatty acids (SCFAs) [31,32]. During aging, reduced abundance of butyrate-producing bacteria may impair mucus production and epithelial energy metabolism, thereby weakening barrier integrity [44,47].

Beneath the mucus layer, epithelial cells are connected by highly regulated intercellular structures known as tight junctions [75,77]. These complexes are composed of proteins including occludin, claudins, and zonula occludens-1 (ZO-1), which collectively control paracellular permeability [77]. Experimental and clinical studies have demonstrated age-related reductions in tight-junction integrity, resulting in increased intestinal permeability [43,75,77]. Inflammatory cytokines, oxidative stress, microbial dysbiosis, and reduced SCFA availability have all been implicated in the disruption of tight-junction architecture [31,43,77].

Importantly, intestinal permeability should not be viewed simply as a local gastrointestinal abnormality [75]. Rather, it represents a systemic biological event capable of reshaping immune function throughout the body. As intestinal barrier integrity progressively declines, the gut becomes increasingly permissive to the translocation of microbial products into the circulation, thereby contributing to the chronic low-grade inflammatory milieu characteristic of aging [9,43,50,75].

Moreover, intestinal permeability exists along a biological continuum rather than as an all-or-none phenomenon and may vary considerably between individuals depending on genetic background, diet, medication use, comorbidities, and microbiome composition [42,53,54,75]. This interindividual heterogeneity further supports the concept that age-associated dysbiosis is better understood as a spectrum of functional microbiome endotypes rather than a single universal “aging microbiome” [20,21,53]. Consequently, distinct functional endotypes may differentially influence barrier integrity, immune remodeling, and ultimately neuroimmune resilience, suggesting that the loss of barrier function reflects the heterogeneous biological trajectories of aging rather than a uniform pathological process [26,53,54].

### 3.2. Microbial Translocation: From Local Dysbiosis to Systemic Inflammation

One of the most significant consequences of intestinal barrier dysfunction is microbial translocation, a process whereby microbial molecules cross the intestinal epithelium and gain access to systemic circulation [75,77]. Although complete bacterial invasion remains uncommon in healthy individuals, the translocation of microbial-derived products occurs with increasing frequency during aging and has been strongly associated with inflammaging [78].

Among the most extensively studied translocated molecules is lipopolysaccharide (LPS), a structural component of the outer membrane of Gram-negative bacteria [79]. Even low circulating concentrations of LPS can activate innate immune pathways through Toll-like receptor 4 (TLR4), triggering the production of pro-inflammatory cytokines including tumor necrosis factor-α (TNF-α), interleukin-6 (IL-6), and interleukin-1β (IL-1β) [9,79].

Additional microbial products capable of entering systemic circulation include peptidoglycan fragments derived from bacterial cell walls and circulating bacterial DNA. These molecules interact with pattern-recognition receptors and further amplify inflammatory signaling pathways [80]. Increasing evidence suggests that chronic exposure to these microbial-associated molecular patterns (MAMPs) contributes to immune activation, endothelial dysfunction, and age-related tissue damage [9,50,80].

Importantly, microbial translocation represents a mechanistic bridge between intestinal dysbiosis and systemic inflammation [24,50,78]. Rather than acting solely as markers of barrier dysfunction, translocated microbial products actively participate in shaping immune responses that influence distant organs, including the brain [17,24,78].

### 3.3. Blood–Brain Barrier Dysfunction During Aging

The blood–brain barrier is a highly selective vascular interface that regulates molecular exchange between the circulation and the central nervous system. It consists of specialized endothelial cells connected by tight junctions, pericytes, astrocytic end-feet, and supporting extracellular matrix components that collectively form the neurovascular unit [81].

Aging is associated with the progressive impairment of BBB integrity [15,81]. Structural and functional alterations include endothelial dysfunction, reduced cerebral blood flow, increased oxidative stress, mitochondrial dysfunction, and the disruption of tight-junction proteins [82]. These changes compromise barrier selectivity and increase permeability to circulating inflammatory mediators [81,82].

Emerging evidence further suggests that age-related degradation of the endothelial glycocalyx may contribute to increased vascular permeability and amplify inflammatory signaling within the neurovascular unit [83]. Because the endothelial glycocalyx constitutes an important regulator of vascular homeostasis, its progressive deterioration may further sensitize the blood–brain barrier to systemic inflammatory insults during aging [81,83].

Recent neuroimaging and biomarker studies have demonstrated that BBB leakage can occur even during normal aging and often precedes measurable cognitive decline [84]. Importantly, BBB dysfunction appears particularly pronounced in brain regions vulnerable to neurodegeneration, including the hippocampus and cortical areas involved in memory processing [84].

Systemic inflammation emerging from the aging gut may further accelerate BBB disruption [9,16,24,78]. Circulating cytokines, LPS, and other microbial-derived molecules promote endothelial activation, oxidative stress, and vascular inflammation, thereby weakening the neurovascular unit [79,81,82]. As BBB integrity declines, inflammatory mediators gain increased access to the central nervous system, creating conditions that favor the chronic activation of resident immune cells.

### 3.4. The Double-Barrier Model: Linking Dysbiosis to Neuroinflammation

Emerging evidence supports a model in which age-associated dysbiosis contributes to interconnected dysfunction of the intestinal and blood–brain barriers rather than affecting these interfaces as independent biological compartments [20,21,43,78,81,85]. Loss of beneficial microbial functions and reduced production of regulatory metabolites may weaken intestinal barrier integrity, thereby facilitating the systemic exposure to microbial products and promoting chronic low-grade inflammation [31,47,54,78]. The resulting inflammatory and metabolic milieu can induce endothelial dysfunction and progressively compromise the neurovascular unit, providing a mechanistic link between intestinal dysbiosis and altered blood–brain barrier (BBB) function [9,81,82,85,86,87].

This coupling is mediated by several complementary signaling pathways. Increased intestinal permeability permits systemic exposure to microbial-associated molecular patterns (MAMPs), including lipopolysaccharide (LPS), peptidoglycan fragments, and bacterial nucleic acids [75,78,79,80]. Among these, LPS activates innate immune signaling through the LBP–CD14–TLR4 receptor complex and downstream MyD88- and TRIF-dependent pathways, leading to NF-κB activation and the increased production of pro-inflammatory mediators such as TNF-α, IL-1β, and IL-6 [79,80]. Persistent exposure to these signals promotes systemic endothelial activation, oxidative stress, and disruption of endothelial tight-junction organization, thereby increasing the susceptibility of the BBB to inflammatory injury [9,81,82,87]. In this context, circulating microbial products and inflammatory mediators constitute an important molecular bridge through which the loss of intestinal containment can influence the neurovascular compartment [78,85,86,88,89].

Loss of protective microbial metabolic signaling represents a complementary component of this process. Short-chain fatty acids (SCFAs), particularly butyrate, contribute to epithelial and immune homeostasis through G-protein-coupled receptor signaling and histone deacetylase inhibition and participate in the regulation of tight-junction integrity and inflammatory transcriptional programs [31,32,47]. Reduced SCFA availability may therefore have consequences extending beyond the intestinal epithelium by altering systemic immune tone and increasing the vulnerability of the neurovascular unit to inflammatory signals [31,51,85,86,90]. Other microbiome-associated metabolic pathways may also contribute. Tryptophan-derived indoles and kynurenine-pathway metabolites can influence immune and neuroimmune signaling, whereas microbiota-modified secondary bile acids participate in host metabolic and immune regulation [28,52,68,69,70,71]. Alterations in these metabolite networks may consequently influence endothelial, immune, and neural homeostasis, although their effects are metabolite-, concentration-, and context-dependent and should not be interpreted as uniformly protective or detrimental [28,52,59,68,69,70,71,90].

Immune-cell signaling provides a further mechanistic connection between the intestinal and cerebral compartments. Persistent microbial stimulation can alter circulating monocyte activity and shape microbiota-conditioned adaptive immune responses, including Th17-associated pathways [24,88,89,91]. Cytokines generated within these networks, including IL-17 together with TNF-α, IL-1β, and IL-6, can amplify endothelial and neuroimmune signaling at CNS-associated vascular and meningeal interfaces [89,91,92,93,94,95]. The BBB should therefore not be considered merely a passive downstream target of peripheral inflammation. Rather, the neurovascular unit constitutes an active interface at which circulating inflammatory, metabolic, and microbial signals interact with endothelial cells, perivascular cells, astrocytes, and resident immune cells, thereby influencing microglial responsiveness and CNS homeostasis [81,85,86,87,91].

**Figure 3:**
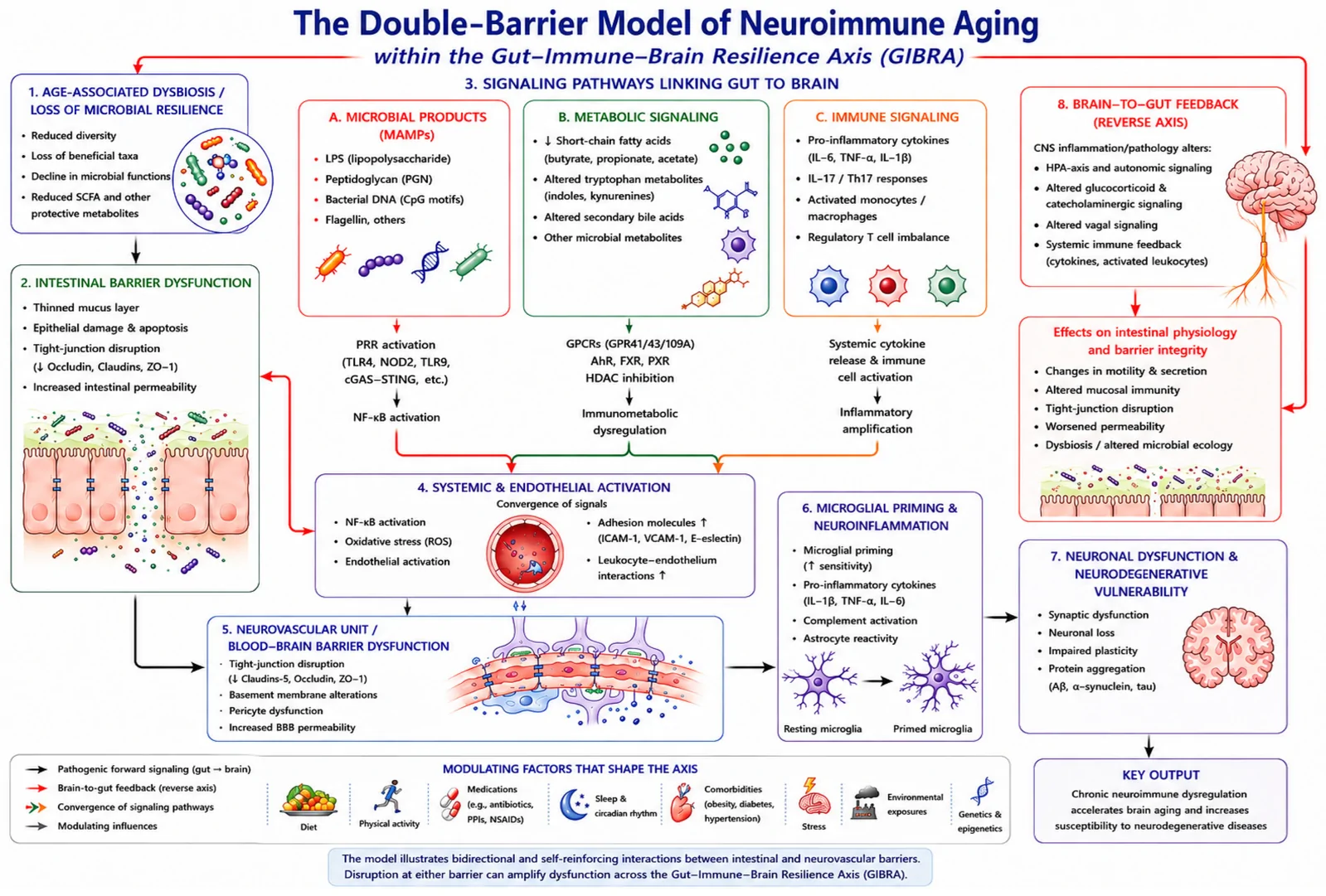
The Double-Barrier Model of Neuroimmune Aging within the Gut–Immune–Brain Resilience Axis (GIBRA). Proposed mechanistic model illustrating how age-associated dysbiosis may contribute to neuroimmune vulnerability through interconnected dysfunction of the intestinal and blood–brain barriers. Impaired intestinal barrier integrity increases systemic exposure to microbial-associated molecular patterns, including lipopolysaccharide (LPS), peptidoglycan fragments, and bacterial nucleic acids, while loss or alteration of microbiome-derived metabolic signals, including short-chain fatty acids (SCFAs), tryptophan-related metabolites, and secondary bile acids, may modify immune and barrier homeostasis. Microbial products, altered metabolite signaling, circulating cytokines, and activated immune-cell pathways converge on systemic and neurovascular endothelial activation, oxidative stress, and blood–brain barrier dysfunction. Increased neurovascular vulnerability facilitates communication between peripheral inflammatory signals and CNS-resident cells, promoting microglial priming, chronic neuroinflammation, neuronal dysfunction, and susceptibility to neurodegenerative processes. Rather than representing a strictly linear gut-to-brain cascade, the model conceptualizes intestinal and neurovascular barrier dysfunction as mechanistically interconnected components of the broader GIBRA network. The model further incorporates a reverse brain-to-gut pathway in which established neurovascular dysfunction and neuroinflammation may influence intestinal physiology, epithelial barrier integrity, and microbial ecology through autonomic, neuroendocrine, and immune signaling. These reciprocal interactions may create a self-reinforcing barrier cycle, although direct validation of the complete bidirectional loop in aging humans remains limited.

Within this expanded Double-Barrier model, intestinal permeability, microbial translocation, altered metabolite signaling, systemic immune activation, endothelial dysfunction, and BBB impairment represent interconnected components of a common biological network rather than a strictly linear sequence (Figure 3) [78,81,85,86]. Their convergence may generate a feed-forward inflammatory environment in which impaired intestinal containment increases systemic exposure to microbial and inflammatory signals, while progressive neurovascular dysfunction enhances CNS susceptibility to these peripheral influences and promotes microglial priming [12,13,14,15,78,81,86,88,89,91]. The aging organism may therefore experience a dual loss of barrier resilience—a more permeable intestinal interface that increases systemic exposure to gut-derived signals and a progressively vulnerable BBB that becomes increasingly responsive to peripheral inflammatory and metabolic perturbations [75,81,84,85].

Importantly, the Double-Barrier model is unlikely to operate exclusively in the gut-to-brain direction. Once neurovascular dysfunction and central neuroinflammation are established, altered CNS output may feed back onto intestinal physiology through autonomic, neuroendocrine, and immune pathways, thereby influencing gastrointestinal motility, secretion, epithelial permeability, mucosal immune activity, and microbial ecology [96,97,98]. Experimental evidence provides direct support for such reverse brain-to-gut signaling. In a murine model of cerebral ischemia, stroke rapidly increased intestinal epithelial cell death and gut permeability, whereas pharmacological sympathetic denervation reduced these abnormalities toward the baseline, identifying sympathetic nervous-system activation as a mechanistic mediator of post-stroke intestinal barrier dysfunction [99]. Complementary experimental studies of traumatic brain injury have demonstrated increased intestinal permeability, reduced tight-junction integrity, and alterations in gut microbial ecology associated with corticotropin-releasing hormone receptor signaling, supporting a neuroendocrine route through which central injury and stress responses may influence intestinal homeostasis [100]. Autonomic regulation appears to be bidirectional: experimental vagal stimulation after traumatic brain injury has been shown to attenuate intestinal barrier dysfunction, further indicating that central neural output can modulate gut epithelial integrity [101]. Together, these findings support a model in which CNS pathology may influence intestinal homeostasis through coordinated sympathetic, vagal, neuroendocrine, and immune mechanisms [96,97,98]. Increased intestinal permeability could, in turn, enhance microbial-product translocation and systemic inflammatory signaling, thereby reinforcing neurovascular dysfunction and neuroinflammation, and creating a potentially self-amplifying brain–gut–brain cycle. However, direct demonstration of this complete reciprocal barrier loop in physiological human aging remains limited; much of the mechanistic evidence for the reverse brain-to-gut arm derives from experimental models of acute CNS injury. The bidirectional component of the Double-Barrier model should therefore be regarded as a biologically plausible and experimentally supported framework that requires confirmation in longitudinal studies of aging and neurodegenerative disease.

Viewed from this perspective, barrier dysfunction is not merely a consequence of aging but a plausible systems-level mechanism through which the age-associated disruption of host–microbiome communication may be translated into chronic neuroimmune vulnerability [43,78,81,85,86]. Nevertheless, the Double-Barrier model should not be interpreted as the sole mechanism underlying gut–brain communication. Neural, autonomic, neuroendocrine, immune, and microbiome-derived metabolic pathways operate in parallel and provide reciprocal routes through which intestinal and CNS dysfunction may influence one another throughout aging [16,17,19,51,52,85,96,98]. Rather than representing competing mechanisms, these pathways constitute complementary components of the integrated gut–immune–brain communication network proposed within the GIBRA framework.

## 4. Immunosenescence and Microglial Priming: The Central Convergence Point

The biological consequences of aging extend far beyond progressive tissue degeneration and the accumulation of cellular damage [8,9,10]. Aging is increasingly recognized as a systemic process characterized by the profound remodeling of immune function that affects virtually every organ system, including the central nervous system [10,11]. Over the past decade, growing evidence has shifted the focus from individual age-associated pathologies toward the concept of neuroimmune aging, a process driven by the interaction between immunosenescence, chronic inflammation, and altered host–microbiome communication [9,10,12,13]. Within this framework, microglial priming has emerged as a potential central convergence point linking age-associated dysbiosis, systemic inflammation, and neurodegenerative vulnerability [14,15,16,78].

### 4.1. Hallmarks of Immunosenescence

Immunosenescence refers to the age-related remodeling of the immune system characterized by the progressive deterioration of immune competence and reduced ability to maintain homeostasis [102]. Although often perceived as a decline in immune function, immunosenescence is better understood as a complex process involving both immune deficiency and immune dysregulation [10,102].

One of the most prominent features of immunosenescence is the progressive reduction of the naïve T-cell pool [103]. Age-associated thymic involution significantly limits the production of new T lymphocytes, resulting in diminished immune diversity and impaired responsiveness to novel antigens [102,103]. Simultaneously, repeated antigenic stimulation throughout life promotes the accumulation of highly differentiated and senescent T-cell populations, many of which exhibit altered cytokine secretion profiles and reduced proliferative capacity [10,102].

Senescent immune cells are not biologically inert. Instead, they actively contribute to systemic inflammation through the secretion of pro-inflammatory mediators collectively referred to as the senescence-associated secretory phenotype (SASP) [104]. This inflammatory environment further reinforces immune dysfunction and contributes to tissue damage across multiple organ systems [9,104].

Importantly, immunosenescence affects both adaptive and innate immunity [10,11,102]. Age-related alterations have been documented in monocytes, macrophages, dendritic cells, natural killer cells, and microglia [11,12,13]. Collectively, these changes result in a chronic state of immune imbalance characterized by reduced immune surveillance, impaired tissue repair, and increased susceptibility to inflammatory diseases [10,11,102].

Recent evidence suggests that the gut microbiome plays an important role in shaping immunosenescence [20,21,30,54]. Age-associated dysbiosis may accelerate immune aging by promoting microbial translocation, chronic antigenic stimulation, and persistent activation of the innate immune pathways [43,50,78]. Consequently, alterations in microbial signaling may not simply accompany immunosenescence but actively contribute to its development [17,31,51,52].

### 4.2. Inflammaging: Chronic Inflammation as a Hallmark of Aging

Closely intertwined with immunosenescence is the phenomenon of inflammaging, defined as a state of chronic, low-grade, sterile systemic inflammation that develops during aging [9,10,102]. Unlike acute inflammation, which serves protective and reparative functions, inflammaging is characterized by the persistent activation of inflammatory pathways in the absence of overt infection [9,102].

Several circulating inflammatory mediators consistently increase with age, including interleukin-6 (IL-6), tumor necrosis factor-α (TNF-α), interleukin-1β (IL-1β), and interferon-γ (IFN-γ) [9,10]. Elevated concentrations of these cytokines have been associated with frailty, cognitive decline, cardiovascular disease, sarcopenia, and increased mortality [9,44,46].

The origins of inflammaging are multifactorial and include cellular senescence, mitochondrial dysfunction, impaired autophagy, accumulation of damage-associated molecular patterns (DAMPs), and persistent activation of innate immune receptors [8,9,102,104]. More recently, age-related dysbiosis and increased intestinal permeability have emerged as important contributors to this inflammatory state [43,50,78].

The aging gut continuously exposes the host to low levels of microbial-derived products such as lipopolysaccharide, peptidoglycan fragments, and bacterial DNA [78,79]. These signals activate pattern-recognition receptors including Toll-like receptors and nucleotide-binding oligomerization domain (NOD)-like receptors, resulting in sustained cytokine production and the amplification of inflammatory signaling networks [9,80].

Emerging evidence further indicates that aging-associated innate immune cells may undergo long-lasting functional reprogramming through mechanisms collectively referred to as trained immunity [105]. Unlike classical immunological memory, trained immunity is mediated by the epigenetic and metabolic reprogramming of innate immune cells, resulting in enhanced inflammatory responsiveness following subsequent stimulation. In the context of aging, persistent microbial signaling and chronic low-grade inflammation may reinforce these adaptive-like innate immune responses, thereby contributing to sustained neuroimmune activation and amplifying inflammaging [50,78,105].

Importantly, inflammaging is increasingly viewed not merely as a consequence of aging but as an active driver of age-related pathology [106]. The brain appears particularly vulnerable to this chronic inflammatory burden, as circulating cytokines and microbial signals can influence blood–brain barrier integrity, microglial activation, and neuronal function [78,81,82,86].

### 4.3. Microglial Biology: Guardians of Brain Homeostasis

Microglia are the resident immune cells of the central nervous system and serve as critical regulators of brain homeostasis. Under physiological conditions, microglia continuously survey the neural microenvironment through highly dynamic cellular processes that monitor neuronal activity, tissue integrity, and potential threats [12,15].

Beyond their classical immune functions, microglia play essential roles in synaptic remodeling, neurogenesis, maintenance of neuronal networks, and the removal of cellular debris [12,15]. During development and adulthood, they contribute to synaptic pruning and support neural plasticity, thereby participating in learning, memory formation, and cognitive adaptation [15].

Homeostatic microglia maintain a finely balanced phenotype that allows for rapid responses to injury while avoiding excessive inflammatory activation [12,15]. This equilibrium is sustained through constant communication with neurons, astrocytes, endothelial cells, and peripheral immune signals [12,15]. Emerging evidence indicates that microbial metabolites, particularly short-chain fatty acids, represent an additional regulatory layer influencing microglial maturation and function [17,31,51].

Experimental studies in germ-free animals have demonstrated profound defects in microglial development and immune competence, highlighting the importance of microbiome-derived signals for maintaining normal microglial physiology [17,19,51]. These observations support the concept that the gut microbiome contributes directly to neuroimmune homeostasis through mechanisms extending far beyond local intestinal effects.

Nevertheless, findings derived from germ-free animal models should be interpreted cautiously because lifelong absence of microbial colonization profoundly alters immune maturation, neurodevelopment, and metabolic homeostasis [107]. Consequently, germ-free models may not fully recapitulate the complex biological processes underlying age-associated dysbiosis in humans and should be viewed primarily as mechanistic rather than translational models [17,51,107].

### 4.4. Microglial Priming: The Final Common Pathway

A growing body of evidence suggests that microglial priming may represent a central convergence point linking age-associated dysbiosis to neurodegenerative vulnerability [12,13,14,16,51].

Microglial priming refers to a state in which microglia acquire heightened sensitivity to inflammatory stimuli while appearing relatively quiescent under basal conditions [14,108]. Primed microglia do not necessarily exhibit overt activation; rather, they remain in a state of increased readiness characterized by exaggerated responses to subsequent immune challenges [14,108].

Several factors associated with aging contribute to the development of this phenotype, including chronic cytokine exposure, oxidative stress, mitochondrial dysfunction, blood–brain barrier disruption, and persistent microbial signaling [9,12,13,14,81,86,106,108]. Age-associated dysbiosis may further exacerbate this process through the reduced production of regulatory metabolites such as butyrate and increased exposure to inflammatory microbial products [31,43,51,78,79].

Accumulating evidence further suggests that microglial priming is accompanied by profound metabolic reprogramming [109]. Homeostatic microglia predominantly rely on oxidative phosphorylation to sustain immune surveillance and tissue maintenance, whereas primed microglia increasingly shift toward glycolytic metabolism [109]. This metabolic transition is associated with the accumulation of intermediary metabolites such as succinate, stabilization of hypoxia-inducible factor-1α (HIF-1α), and the enhanced production of pro-inflammatory cytokines, particularly interleukin-1β (IL-1β) [109,110]. These observations indicate that metabolic remodeling represents an integral component of microglial activation rather than merely a consequence of inflammation.

Functionally, primed microglia display an increased expression of inflammatory receptors, enhanced production of cytokines, exaggerated activation of NF-κB signaling pathways, and heightened responsiveness to secondary insults [14,108]. Consequently, stimuli that would normally evoke modest immune responses may trigger disproportionately strong neuroinflammatory reactions in the aging brain [14,108]. Persistent microbial signaling and mitochondrial dysfunction may also promote activation of the NLRP3 inflammasome, thereby facilitating IL-1β maturation and further amplifying chronic neuroinflammatory responses during aging [80,110].

The concept of microglial priming provides a mechanistic explanation for why aging is associated with increased susceptibility to neurodegenerative disorders [12,13,14,108]. Rather than acting as passive bystanders, primed microglia may serve as amplifiers of systemic inflammatory signals, translating peripheral immune disturbances into chronic neuroinflammation [14,16,86,108]. This process ultimately promotes synaptic dysfunction, neuronal injury, and progressive cognitive decline [12,13,14,15,23,108].

**Figure 4:**
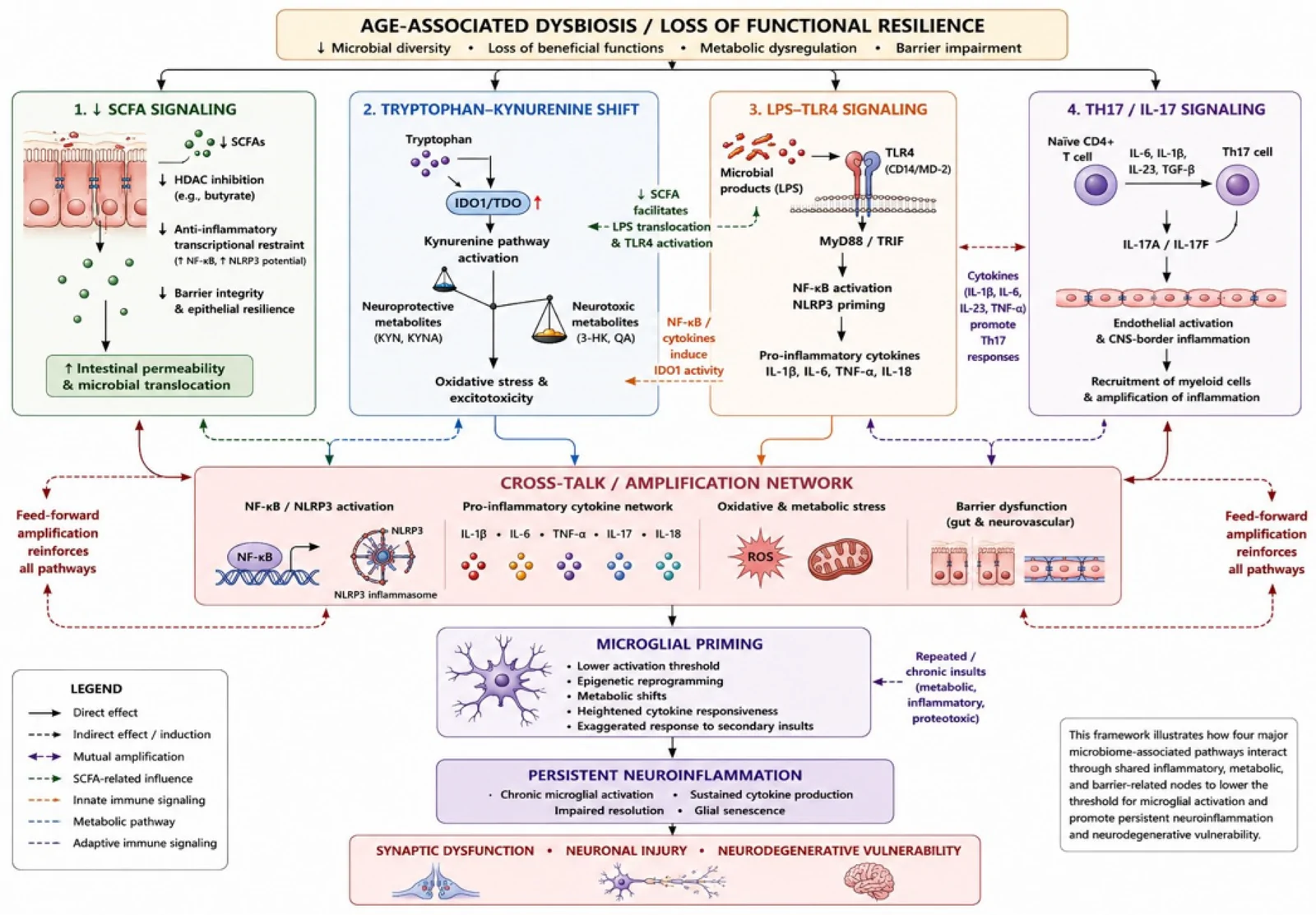
Integrated cross-talk network driving microglial priming during neuroimmune aging. Proposed mechanistic framework illustrating how age-associated dysbiosis and loss of microbial functional resilience may promote microglial priming through four interconnected gut–immune–brain signaling pathways. Reduced SCFA signaling may weaken epithelial barrier resilience and anti-inflammatory and epigenetic restraint, thereby facilitating microbial translocation and innate immune activation [31,42]. Dysregulation of tryptophan–kynurenine metabolism may alter the balance of neuroactive metabolites and contribute to oxidative and excitotoxic stress [17,19,43]. In parallel, microbial products such as LPS activate TLR4-dependent NF-κB signaling and NLRP3 inflammasome priming, promoting pro-inflammatory cytokine production [54,55,69], whereas Th17/IL-17-associated responses may amplify inflammatory signaling at the intestinal, systemic, and CNS-associated vascular interfaces [24,71]. Importantly, these pathways are not biologically independent. Loss of SCFA-mediated barrier and immune restraint may increase the context for microbial translocation and innate immune activation; LPS- and cytokine-driven inflammatory signaling may modulate IDO1-dependent kynurenine metabolism; and innate cytokine networks may interact with Th17/IL-17 responses. These processes converge through shared inflammatory, metabolic, oxidative, and barrier-related nodes, including NF-κB/NLRP3 activation, pro-inflammatory cytokine signaling, oxidative and metabolic stress, and intestinal and neurovascular barrier dysfunction. Their reciprocal amplification may lower the threshold for microglial activation and enhance responsiveness to subsequent inflammatory, metabolic, or proteotoxic insults, thereby promoting persistent neuroinflammation, synaptic dysfunction, neuronal injury, and increased neurodegenerative vulnerability. The proposed network should be interpreted as an integrative mechanistic framework rather than as a strictly linear or universally established sequence of events. Abbreviations: AhR, aryl hydrocarbon receptor; CNS, central nervous system; HDAC, histone deacetylase; IDO1, indoleamine 2,3-dioxygenase 1; IL, interleukin; KYN, kynurenine; KYNA, kynurenic acid; LPS, lipopolysaccharide; MD-2, myeloid differentiation factor 2; MyD88, myeloid differentiation primary response 88; NF-κB, nuclear factor kappa B; NLRP3, NOD-, LRR- and pyrin domain-containing protein 3; QA, quinolinic acid; ROS, reactive oxygen species; SCFA, short-chain fatty acid; TDO, tryptophan 2,3-dioxygenase; TGF-β, transforming growth factor beta; Th17, T helper 17; TLR4, Toll-like receptor 4; TNF-α, tumor necrosis factor alpha; TRIF, TIR-domain-containing adapter-inducing interferon-β.

Taken together, current evidence suggests that neuroimmune aging is driven not by isolated inflammatory pathways but by coordinated interactions among immune remodeling, metabolic reprogramming, microbial signaling, and innate immune memory, all of which converge on microglial priming as a central integrative mechanism (Figure 4) [91,105,109,111]. Within this framework, immunosenescence, inflammaging, and microglial priming represent interconnected manifestations of a common biological process, while microbial translocation, barrier dysfunction, and loss of beneficial microbial signaling act as complementary upstream drivers. The following sections examine the molecular pathways through which age-associated dysbiosis orchestrates these interconnected mechanisms and ultimately promotes neuroinflammatory vulnerability.

## 5. Mechanistic Drivers of Microglial Priming

Building upon the concepts introduced in the previous sections, current evidence indicates that age-associated dysbiosis promotes microglial priming through multiple converging microbial, metabolic, and immune pathways rather than through a single pathogenic mechanism. Together, these interconnected processes position microglial priming as a central mechanistic hub linking gut dysbiosis to neuroimmune aging (Figure 4).

### 5.1. Loss of SCFA Signaling and Epigenetic Deregulation

Among the microbiome-derived metabolites, butyrate has emerged as one of the principal regulators of neuroimmune homeostasis owing to its immunomodulatory and epigenetic properties [31,32,51]. Beyond serving as an energy substrate for colonocytes, butyrate functions as an endogenous inhibitor of histone deacetylases (HDACs), thereby modulating chromatin accessibility, inflammatory gene expression, oxidative stress responses, and cellular differentiation [31,32,47].

Experimental studies in germ-free and antibiotic-treated animal models indicate that microbiota-derived SCFAs are essential for normal microglial maturation and immune competence. Absence or depletion of microbial signals results in immature microglial phenotypes characterized by altered morphology, impaired immune responses, and defective homeostatic function. Nevertheless, findings from germ-free animal models should be interpreted cautiously because the lifelong absence of microbial colonization profoundly alters immune and neurodevelopmental maturation and may not fully recapitulate age-associated dysbiosis in humans [17,19,51,107].

Unhealthy aging and frailty have frequently been associated with a reduced abundance of selected butyrate-producing taxa, including *Faecalibacterium prausnitzii*, *Roseburia* spp., and *Eubacterium* spp. [20,21,44,46,54]. However, reduced SCFA availability should not be interpreted as an age-specific metabolic signature, because SCFA production and systemic exposure are jointly shaped by microbial community structure and functional capacity, dietary substrate availability, particularly fermentable fiber intake, intestinal transit, medication exposure, physical activity, comorbidities, and host absorptive and metabolic processes [31,42,54,66,67]. Chronological age therefore represents only one component of a broader set of determinants influencing SCFA availability. Reduced SCFA signaling, irrespective of its underlying determinants, may weaken epigenetic regulation within the peripheral immune cells and microglia. Diminished HDAC inhibition may favor pro-inflammatory transcriptional programs, increased cytokine production, and heightened sensitivity to inflammatory stimuli. Through these mechanisms, impaired SCFA signaling may facilitate the transition from homeostatic microglial function toward a primed, inflammation-sensitive state (Figure 4) [31,47,51].

### 5.2. Kynurenine Pathway Dysregulation

Chronic inflammation profoundly reshapes tryptophan metabolism through the activation of indoleamine 2,3-dioxygenase 1 (IDO1), the rate-limiting enzyme of the kynurenine pathway [68,69,70,106]. Inflammatory cytokines, particularly interferon-γ (IFN-γ) and tumor necrosis factor-α (TNF-α), induce IDO1 activity, diverting tryptophan metabolism away from serotonin synthesis toward kynurenine production and thereby altering the balance of neuroactive metabolites [68,70,71].

The kynurenine pathway generates both neuroprotective and neurotoxic metabolites [69]. Kynurenic acid exerts neuroprotective effects by modulating glutamatergic neurotransmission and limiting excitotoxicity, whereas quinolinic acid acts as a potent N-methyl-D-aspartate (NMDA) receptor agonist that promotes oxidative stress, mitochondrial dysfunction, excitotoxic neuronal injury, and microglial activation [69].

Growing evidence indicates that age-associated dysbiosis influences kynurenine metabolism through both direct microbial effects and indirect immune-mediated mechanisms [17,19,52,70]. Reduced production of anti-inflammatory microbial metabolites together with persistent immune activation favors a metabolic shift toward the neurotoxic branch of the pathway [31,52,70]. Elevated kynurenine-to-tryptophan ratios and increased quinolinic acid concentrations have been associated with cognitive decline, neurodegenerative disorders, and systemic inflammatory burden [68,69]. Consequently, dysregulation of the kynurenine pathway represents an important mechanistic interface linking gut microbial dysfunction to neuroimmune aging and microglial priming [17,52,68,70].

### 5.3. LPS Endotoxemia and TLR4 Activation

Following translocation across the aging intestinal barrier, circulating lipopolysaccharide (LPS) interacts with Toll-like receptor 4 (TLR4) expressed on monocytes, macrophages, endothelial cells, and microglia [78,79,80,86]. Activation of TLR4 initiates MyD88-dependent intracellular signaling, leading to the activation of nuclear factor kappa B (NF-κB) and the transcription of multiple pro-inflammatory mediators [80].

Sustained NF-κB activation promotes the production of pro-inflammatory cytokines, chemokines, and reactive oxygen species, thereby reinforcing the chronic inflammatory milieu characteristic of aging [9,80,106,110]. In parallel, NF-κB provides the transcriptional priming signal required for the increased expression of NLRP3 and pro-IL-1β, thereby licensing subsequent inflammasome assembly and activation [110,112].

Activation of the NLRP3 inflammasome promotes the maturation and secretion of interleukin-1β (IL-1β) and interleukin-18 (IL-18), further amplifying neuroinflammatory signaling and facilitating microglial priming [110,112]. Experimental studies indicate that prolonged exposure to ultra-low LPS concentrations can induce a trained, memory-like microglial phenotype characterized by enhanced inflammatory responses to subsequent stimulation, whereas higher priming doses may instead promote endotoxin tolerance [113]. Consequently, chronic microbial translocation may transform the aging gut into a sustained source of innate immune activation, linking intestinal barrier dysfunction to progressive neuroimmune dysregulation. Within the integrated model presented in Figure 4, microbial translocation provides a mechanistic link between barrier dysfunction and the activation of innate immune pathways that ultimately converge on microglial priming [92,94,95,114,115].

### 5.4. Gut-Conditioned Th17 Responses and Meningeal IL-17 Signaling

Beyond microbial metabolites and innate immune activation, adaptive immune responses have emerged as additional mediators of gut–brain communication [24,28,91]. The intestinal mucosa serves as a major site of T-cell education, where microbial signals influence differentiation and the functional polarization of immune cell populations [28,31,32,47,91].

Age-associated dysbiosis and persistent inflammatory signaling may promote the expansion of pathogenic T-helper 17 (Th17) cell populations characterized by the increased production of interleukin-17 (IL-17), granulocyte-macrophage colony-stimulating factor (GM-CSF), and other pro-inflammatory mediators [30,43,47,50,91,92]. Experimental evidence indicates that encephalitogenic Th17 cells can traffic between intestinal and extraintestinal immune compartments, while IL-17-producing lymphocytes residing in or recruited to CNS-associated border tissues may modulate neuroinflammatory responses [91,92,93,94].

Particular attention has focused on the meninges, which are increasingly recognized as an active immunological interface linking peripheral and central immune responses [93,94]. Experimental studies demonstrate that the meninges harbor resident IL-17-producing γδ T cells capable of modulating neuronal and glial function. Under inflammatory or age-related conditions, changes in the abundance, activation state, or cytokine output of IL-17-producing lymphocytes may alter signaling within the CNS border tissues and influence neuroinflammatory responses [93,94,95].

Although the precise cellular origins and trafficking routes remain incompletely defined, microbiota-conditioned Th17 responses and IL-17-producing lymphocytes within CNS-associated border tissues represent plausible complementary pathways through which dysbiosis may influence neuroimmune function [91,92,93,94]. By sustaining chronic cytokine exposure and reinforcing inflammatory signaling networks, these adaptive immune responses may further promote the transition toward maladaptive microglial activation [12,13,14,91,95,108].

Rather than representing independent pathogenic mechanisms, loss of SCFA signaling, dysregulated kynurenine metabolism, LPS–TLR4 activation, and Th17/IL-17 signaling are likely to interact through shared inflammatory, metabolic, and epigenetic nodes that collectively lower the threshold for microglial activation [31,51,70,78,91,92,93,94,95,110,112]. Reduced SCFA signaling may diminish HDAC-dependent restraint of inflammatory transcription and weaken epithelial barrier integrity, thereby increasing the susceptibility to microbial translocation and LPS-driven innate immune activation [31,47,51,78]. LPS–TLR4 signaling, in turn, activates NF-κB and promotes the production of IL-1β, IL-6, and TNF-α, cytokines that reinforce systemic inflammation and can influence adaptive immune polarization and neurovascular inflammatory signaling [80,91,110,112]. The resulting inflammatory milieu may simultaneously enhance the IDO1-dependent diversion of tryptophan toward the kynurenine pathway, thereby coupling innate immune activation to altered neuroactive metabolite balance [68,69,70,71,106]. Th17/IL-17 signaling may further amplify endothelial and CNS-border inflammation and reinforce cytokine networks that sustain microglial responsiveness [91,92,93,94,95]. These pathways therefore converge not only directly on microglia, but also indirectly reinforce one another through shared NF-κB/NLRP3 activation, cytokine signaling, oxidative and metabolic stress, altered barrier integrity, and loss of anti-inflammatory metabolic restraint. The resulting network may establish a feed-forward state in which microbial translocation and inflammatory signaling progressively increase microglial sensitivity to subsequent metabolic, immune, and proteotoxic insults, promoting the transition from reversible priming toward persistent neuroinflammatory activation (Figure 4).

## 6. From Microglial Priming to Neurodegeneration

Microglial priming is increasingly recognized as a critical mechanistic link between age-associated immune dysfunction and neurodegenerative disease [12,13,14,108,116]. While aging itself is accompanied by low-grade neuroinflammation, primed microglia amplify the effects of subsequent inflammatory, metabolic, and proteotoxic insults, thereby creating a permissive environment for neuronal dysfunction and progressive neurodegeneration [9,12,13,14,106,108]. In this context, age-associated dysbiosis, barrier dysfunction, and chronic peripheral inflammation do not directly cause neurodegenerative diseases but may lower the threshold for pathological processes that ultimately culminate in cognitive decline and neurological impairment [16,19,23,24,78,86,91,116].

Importantly, microglial priming provides a biologically plausible explanation for why aging represents the strongest risk factor for most neurodegenerative disorders [1,2,8,12]. Rather than acting as passive bystanders, primed microglia actively shape disease progression through persistent cytokine production, the impaired clearance of protein aggregates, oxidative stress generation, and disruption of neuronal homeostasis [12,13,14,15,108,117]. Consequently, many age-related neurological disorders may be viewed through the broader framework of neuroimmune aging.

### 6.1. Alzheimer’s Disease

Alzheimer’s disease (AD) is the most common neurodegenerative disorder worldwide and remains the leading cause of dementia among older adults [2,3]. Traditionally, the disease has been characterized by two neuropathological hallmarks: extracellular deposition of amyloid-β (Aβ) plaques and the intracellular accumulation of hyperphosphorylated tau protein forming neurofibrillary tangles [6,7,118]. However, growing evidence indicates that these pathological features alone cannot fully explain disease onset, progression, or clinical heterogeneity.

Neuroinflammation has emerged as a central component of Alzheimer’s disease pathogenesis [12,13,108,116,117,118]. Genome-wide association studies have identified numerous AD susceptibility genes involved in innate immune signaling and microglial function, including TREM2, CD33, CR1, and ABI3, highlighting the importance of immune pathways in disease development [119]. These findings have shifted the field from a purely amyloid-centered model toward a more integrated neuroimmune framework [117,118,119].

Microglia play a dual role in Alzheimer’s disease [117,120]. During early or selected phases of pathology, microglia may restrict tissue damage through plaque compaction, containment of toxic amyloid species, and phagocytic clearance [117,120]. However, prolonged exposure to inflammatory stimuli may induce a primed phenotype characterized by excessive cytokine production, impaired phagocytic function, and the chronic activation of inflammatory signaling pathways [12,13,14,108,116,117]. Under these conditions, microglia become less efficient at clearing amyloid deposits while simultaneously promoting neuronal injury [117,121].

Recent studies suggest that gut-derived inflammatory signals may contribute to this transition [86,91,116,118]. Increased intestinal permeability, microbial translocation, and chronic endotoxemia have been associated with elevated circulating inflammatory mediators capable of influencing microglial activity [75,78,79,86,91,116]. Experimental models have demonstrated that microbial dysbiosis can accelerate amyloid deposition, enhance neuroinflammation, and worsen cognitive performance [122,123]. Although reductions in several butyrate-producing taxa are frequently reported in AD cohorts, the effects of SCFAs on microglial function and amyloid pathology appear context-dependent, varying according to metabolite composition, concentration, disease stage, and experimental model [31,51,124].

Nevertheless, evidence linking gut dysbiosis to Alzheimer’s disease remains heterogeneous [118,125,126]. Differences in study populations, sequencing methodologies, dietary habits, medication use, disease stage, and geographic variability contribute to inconsistent microbial signatures reported across human cohorts [67,118,125,126]. Consequently, although functional alterations in microbial signaling appear increasingly reproducible, a universal Alzheimer’s disease-associated microbiome has not been identified [53,118,125,126].

Tau pathology may also be influenced by neuroimmune mechanisms. Microglial inflammatory signaling can influence tau phosphorylation, seeding, and interregional propagation through pathways involving NF-κB, cytokine release, inflammasome activation, and altered processing of extracellular tau; however, selected plaque-associated microglial states may also restrict tau seeding and spread [127,128,129]. Thus, microglial priming may function as a common amplifier linking peripheral inflammation, amyloid pathology, tau pathology, and progressive neurodegeneration [108,116,127,128,129].

### 6.2. Parkinson’s Disease

Parkinson’s disease (PD) is the second most common neurodegenerative disorder and is characterized by the progressive degeneration of dopaminergic neurons within the substantia nigra pars compacta [4,5,130]. The pathological hallmark of PD is the accumulation of misfolded α-synuclein aggregates that form Lewy bodies and Lewy neurites throughout the nervous system [130].

In recent years, increasing attention has been directed toward the gut as a potential site of disease initiation [131,132]. This concept, commonly framed as the gut-origin or body-first hypothesis and conceptually related to the Braak staging model, proposes that in a subset of patients, pathological α-synuclein may arise within the enteric or peripheral autonomic nervous system and subsequently propagate toward the brain through interconnected neural pathways, including the vagus nerve [131,132,133].

Several observations support this hypothesis. Gastrointestinal symptoms, particularly constipation, frequently precede motor manifestations by years or even decades [131,134]. Pathological or phosphorylated α-synuclein has been detected in gastrointestinal and other peripheral neural tissues in some patients with established or prodromal synucleinopathy; however, variable sampling sites, staining protocols, specificity, and diagnostic sensitivity currently limit its use as a definitive biomarker of gut-origin disease [131,134]. Age-associated dysbiosis, increased intestinal permeability, and chronic inflammation may contribute to the initiation and propagation of these pathological processes [20,21,43,50,78,91,131].

Although the gut-origin hypothesis has gained substantial experimental support, its applicability to all patients with Parkinson’s disease remains debated [131,135,136]. Emerging imaging and neuropathological evidence supports heterogeneous disease trajectories, including body-first and brain-first patterns, which may represent biologically distinct but partially overlapping subtypes rather than mutually exclusive mechanisms [135,136].

Microglia again appear to occupy a central position within disease progression [12,13,14,108,137,138]. Extracellular monomeric, oligomeric, and fibrillar α-synuclein species can engage microglial pattern-recognition receptors, particularly TLR2, and depending on the α-synuclein assembly and experimental context, TLR4 or TLR5, thereby activating NF-κB-dependent transcription and the NLRP3 inflammasome [137,138,139]. Primed microglia respond to these stimuli with exaggerated production of inflammatory mediators, thereby amplifying neuronal stress and accelerating dopaminergic neurodegeneration [14,108,137,138,139,140].

Experimental studies in α-synuclein-overexpressing mice have demonstrated that germ-free conditions or antibiotic-mediated microbiota depletion can attenuate microglial activation, α-synuclein pathology, and motor impairment, whereas microbial recolonization can restore or exacerbate these phenotypes [141]. Microbial metabolites, including SCFAs, bile-acid derivatives, tryptophan metabolites, and microbially modified dietary compounds, may influence α-synuclein homeostasis, mitochondrial function, oxidative stress, immune activation, and intestinal barrier integrity; however, their effects are metabolite-, dose-, and context-dependent and should not be interpreted as uniformly protective or pathogenic [17,28,31,52,91,131,141,142,143].

Collectively, these findings support the notion that Parkinson’s disease may represent a disorder of both protein misfolding and neuroimmune dysregulation, with microglial priming serving as a key mediator linking peripheral microbial signals to central neurodegenerative processes [91,108,131,137,141].

### 6.3. Beyond AD and PD: Vascular Cognitive Impairment and Frontotemporal Dementia

The potential relevance of the Gut–Immune–Brain Resilience Axis may extend beyond Alzheimer’s and Parkinson’s diseases to other age-related neurodegenerative and neurovascular disorders, although the maturity of evidence differs substantially across conditions. In cerebral small vessel disease (CSVD), emerging human multi-omics studies provide particularly relevant support for interactions among gut microbial alterations, circulating and fecal metabolites, neurovascular dysfunction, and cognitive impairment. Integrated microbiome–metabolome–neuroimaging analyses have linked specific microbial and metabolic signatures with alterations in gray- and white-matter integrity, intrinsic brain activity, structural network organization, and cognitive performance [144]. Complementary metagenomic and metabolomic evidence in arteriosclerotic CSVD has identified alterations involving microbial lipopolysaccharide biosynthesis and phenylalanine–tyrosine metabolism, suggesting potential connections between microbial functional activity, systemic inflammatory and metabolic signaling, and cerebral vascular pathology [145]. Gut microbial composition has also been associated with neurovascular coupling and cognitive dysfunction in patients with CSVD, further supporting the neurovascular unit as a plausible interface linking peripheral microbial signals with brain function [146]. This interpretation is consistent with independent evidence relating integrated neurovascular-unit dysfunction, including blood–brain barrier leakage and microvascular abnormalities, to cognitive performance in CSVD [147]. Collectively, these findings suggest that vascular cognitive impairment may represent an important extension of the GIBRA framework in which microbial–metabolic and inflammatory disturbances converge particularly strongly on endothelial and neurovascular resilience [148]. Evidence for frontotemporal dementia (FTD) is considerably less developed and should therefore be interpreted more cautiously. Nevertheless, experimental evidence from *C9orf72*-deficient models demonstrates that the intestinal microbial environment can modulate systemic inflammation, neural inflammation, and microglial responses, providing mechanistic support for gene–microbiome–immune interactions relevant to the FTD/ALS spectrum [149]. Thus, rather than implying a uniform microbiome signature across neurodegenerative diseases, these observations support a transdiagnostic but disease-contextualized interpretation of GIBRA, in which shared microbial–immune–metabolic mechanisms may converge on different dominant host vulnerabilities—particularly neurovascular dysfunction in vascular cognitive impairment and genetically conditioned immune dysregulation in selected FTD-related contexts [150]. However, direct longitudinal human microbiome evidence in FTD remains limited, and disease-specific causal relationships require prospective validation.

### 6.4. Cognitive Decline, Frailty, and Loss of Neuroimmune Resilience

Not all consequences of neuroimmune aging manifest as clinically defined neurodegenerative diseases. Cognitive decline and frailty are highly prevalent among older adults and frequently develop in the absence of overt Alzheimer’s or Parkinson’s disease pathology. Increasing evidence suggests that these conditions may also be influenced by chronic neuroinflammatory processes and altered gut–brain communication [8,10,12,15,20,21,44,46,51].

Cognitive aging is characterized by gradual impairments in memory, executive function, processing speed, and attention [8,10,15]. Although multiple factors contribute to these changes, chronic activation of innate immune pathways has emerged as a significant determinant of age-related cognitive dysfunction. Elevated concentrations of inflammatory cytokines, including IL-6 and TNF-α, have consistently been associated with poorer cognitive performance and accelerated cognitive decline [114].

Microglial priming may play a particularly important role in this process. Persistent low-grade activation of microglia promotes synaptic remodeling abnormalities, impaired synaptic plasticity, mitochondrial dysfunction, and reduced neuronal adaptability [12,15,109,110,117]. Over time, these alterations compromise neural network integrity and diminish cognitive reserve.

Frailty represents another manifestation of systemic biological aging characterized by reduced physiological resilience and increased vulnerability to stressors. Interestingly, frailty and cognitive decline frequently coexist, suggesting shared mechanistic pathways [44,45,46,48,49,50,53,54,151]. Both conditions have been associated with chronic inflammation, dysbiosis, reduced microbial diversity, and altered metabolite production [44,45,46,50,114].

From a systems-biology perspective, frailty may be viewed as a whole-organism consequence of diminished resilience across interconnected physiological networks, including the immune system, gut microbiome, vasculature, and brain [115,151,152,153]. Within this framework, microglial priming serves not only as a marker of neuroimmune aging, but also as a mediator of declining adaptive capacity.

Taken together, current evidence suggests that age-associated dysbiosis, barrier dysfunction, immunosenescence, and chronic inflammation converge on microglial priming as a central neuroimmune mechanism [115,151,152,153]. Whether manifested as Alzheimer’s disease, Parkinson’s disease, or more subtle forms of cognitive decline and frailty, these conditions share common biological pathways characterized by impaired resilience, persistent neuroinflammation, and progressive loss of homeostatic control [152,153,154,155]. Understanding these shared mechanisms may provide new opportunities for interventions aimed at preserving brain health and promoting healthy aging.

### 6.5. Strengths and Limitations of Current Human and Experimental Evidence

Interpretation of the current evidence should nevertheless remain cautious. Although experimental models have substantially advanced the understanding of gut–immune–brain interactions, each approach possesses important methodological limitations. Germ-free animal models profoundly alter immune maturation and neurodevelopment throughout life, making them imperfect surrogates for age-associated dysbiosis in humans [107]. Likewise, fecal microbiota transplantation (FMT) studies vary considerably according to donor characteristics, recipient age, microbial engraftment efficiency, housing conditions, and experimental protocols, which may contribute to inconsistent findings across studies [107,156]. Moreover, important differences in microbiome composition, immune architecture, lifespan, and environmental exposures between murine models and humans limit the direct translation of experimental observations into clinical practice [157]. Consequently, the integration of complementary evidence across human cohorts, experimental models, and multi-omics approaches will be essential for testing and refining the GIBRA framework and defining clinically meaningful biomarkers of neuroimmune resilience (Table 2) [16,19,155].

Emerging longitudinal human evidence has begun to strengthen the temporal dimension of the microbiome–cognition relationship, although it remains insufficient to establish causality. In a longitudinal cohort of 260 community-dwelling adults aged ≥60 years spanning healthy cognition, mild cognitive impairment, and dementia, metagenomic sequencing identified microbial functional pathways related to the urea cycle, polyamine synthesis, and methionine and cysteine metabolism that were associated with poorer cognitive performance, providing functional rather than exclusively taxonomic evidence of an association between the gut microbiome and cognitive impairment [158]. Complementary prospective evidence from three large U.S. cohorts comprising 112,753 participants showed that altered bowel-movement frequency was associated with subsequent subjective and objective cognitive outcomes over a median follow-up of up to four years; shotgun metagenomic profiling in a substantially smaller subset further linked these phenotypes to overall microbial configuration and specific microbial species, including the depletion of butyrate-producing taxa [159]. In addition, a prospective cohort of 746 older adults with overweight/obesity and metabolic syndrome demonstrated that a baseline Mediterranean diet-related gut microbial signature was associated with cognitive trajectories assessed repeatedly over six years [160]. Human functional evidence is further supported by a study of 159 long-term-care residents, in whom severe cognitive impairment was associated with increased microbial capacity for methanogenesis and reduced capacity for the synthesis of short-chain fatty acids, glutamate, γ-aminobutyric acid, and amino acids involved in lysosomal function, independently of age, sex, antibiotic exposure, and diet [161]. However, the latter study was cross-sectional and therefore cannot establish temporal ordering, while the prospective studies also have important limitations: microbiome profiling was performed in substantially smaller subsets or primarily at the baseline, study populations were clinically selected in some cohorts, and repeated functional metagenomic or metabolomic measurements were generally unavailable. Thus, current human evidence supports prospective and longitudinal associations between microbiome-related features and subsequent cognitive trajectories, but does not yet demonstrate that loss of microbial functional capacity precedes, or causally drives, neuroinflammation and neurodegeneration. Resolving this directionality will require large prospective cohorts incorporating repeated microbiome functional profiling and metabolomics alongside longitudinal cognitive phenotyping, systemic and CNS-associated inflammatory biomarkers, and where feasible, neuroimaging, while rigorously accounting for major modifiers including age, frailty, diet, medication exposure, intestinal transit, comorbidity burden, and evolving neurological disease.

## 7. Multi-Omics Approaches and Causal Inference

One of the major challenges in microbiome research is distinguishing biological association from causation [19,156,162]. Over the past decade, numerous studies have reported links between gut microbial composition and aging-related neurological disorders [16,20,21,23,24,44,53,54]. However, the presence of microbial alterations does not necessarily imply that these changes directly contribute to disease pathogenesis [156,157]. A central question therefore remains: which microbial signals actively drive neuroimmune aging, and which merely reflect underlying disease processes [91,156,162].

Traditional microbiome studies have relied predominantly on taxonomic profiling, generating valuable insights into microbial diversity and composition [20,21,22,29,53]. Yet, microorganisms exert biological effects through their functional activities rather than their taxonomic identity alone [17,26,31,163]. Consequently, contemporary research is increasingly shifting from descriptive microbiome analysis toward integrated multi-omics approaches capable of capturing microbial function, host responses, and inter-organ communication networks [162,163,164,165].

**Figure 5:**
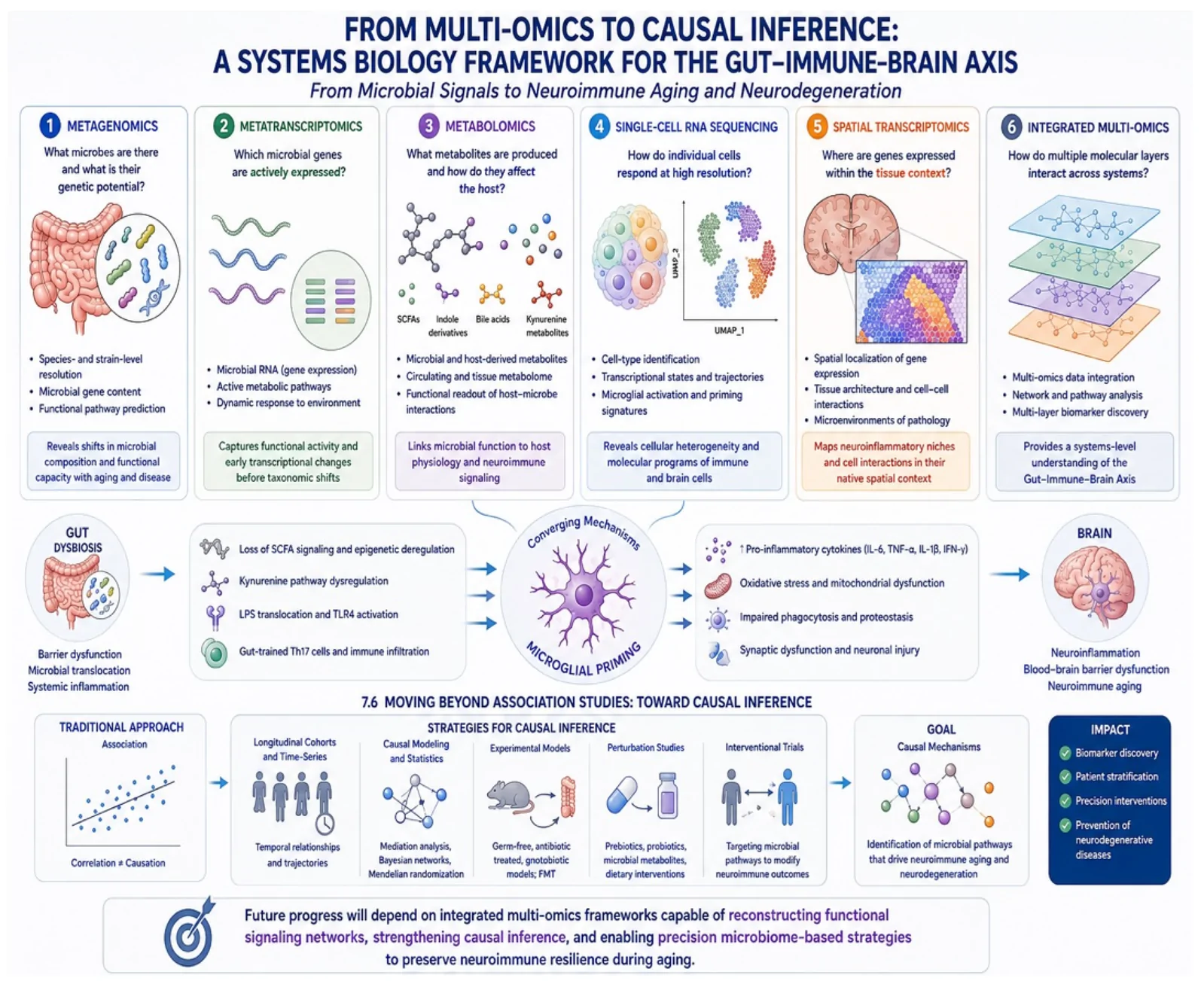
Multi-Omics Integration for Causal Inference in Neuroimmune Aging. Integrated multi-omics approaches provide complementary layers of biological information spanning microbial composition, transcriptional activity, metabolite production, cellular heterogeneity, and spatial tissue organization. Their combined analysis enables the systems-level reconstruction of the gut–immune–brain axis and facilitates the identification of functional microbial pathways, microglial priming mechanisms, and candidate causal drivers of neuroimmune aging. When integrated with longitudinal sampling, host genetic information, experimental perturbation, and advanced computational modeling, these approaches may strengthen causal inference, improve biomarker discovery and patient stratification, and support the development of precision microbiome-based interventions [162,164,165,166].

Recent advances in high-throughput technologies have enabled unprecedented characterization of the gut–immune–brain axis at multiple biological levels [162,163,164,165]. By integrating metagenomics, metatranscriptomics, metabolomics, single-cell transcriptomics, and spatially resolved molecular profiling, investigators are beginning to identify the mechanistic pathways through which microbial signals influence neuroimmune aging (Figure 5) [46,51,52,162,163,164,165].

### 7.1. Metagenomics

Metagenomic sequencing has fundamentally transformed microbiome research by allowing for the comprehensive characterization of microbial communities without the need for culture-based methods [163,167]. Unlike targeted 16S ribosomal RNA sequencing, shotgun metagenomics provides species- and strain-level resolution while simultaneously identifying microbial genes and metabolic pathways [163,167].

In the context of aging, metagenomic studies have demonstrated age-associated changes in microbial richness and diversity, the depletion of selected butyrate-producing taxa, and the enrichment of potentially pro-inflammatory or disease-associated microorganisms, although these patterns vary substantially according to health status, geography, diet, medication exposure, and study design [20,21,22,46,53,54,56]. More importantly, metagenomic analyses have revealed age-associated shifts in microbial functional capacity, including alterations in pathways involved in short-chain fatty acid biosynthesis, tryptophan and amino-acid metabolism, bile-acid transformation, cell-wall biosynthesis, and other inflammatory or metabolic functions [28,31,46,52,53,54,56,57].

These observations suggest that microbial function may be more informative for host physiology than microbial composition alone [26,163]. Two individuals may harbor taxonomically distinct microbial communities while maintaining partially overlapping gene repertoires or metabolic outputs, reflecting functional redundancy within the human microbiome [26,55]. This emphasizes the importance of integrating functional metagenomic profiles with metatranscriptomic, metabolomic, and host-derived molecular data rather than relying exclusively on taxonomic abundance [162,163,167].

### 7.2. Metatranscriptomics

While metagenomics identifies microbial genetic and functional potential, it cannot determine which microbial genes are actively transcribed under a given physiological condition [163,167,168]. Metatranscriptomics addresses this limitation by quantifying community-wide microbial RNA transcripts, thereby providing a dynamic representation of microbial gene expression and functional activity [163,168,169].

Metatranscriptomic profiles can vary independently of, and often more rapidly than, relatively stable taxonomic and metagenomic profiles [168,169]. Consequently, this approach provides insight into dynamic microbial responses to dietary exposures, inflammation, host physiology, environmental perturbations, and other temporally variable conditions [163,168,169,170].

Available evidence suggests that aging is associated with alterations in both microbial community structure and microbiome functional activity, including pathways related to metabolite production, stress adaptation, intestinal barrier regulation, and host–microbe communication [20,21,49,50,54,163,168,171]. However, direct longitudinal evidence showing that microbial transcriptional reprogramming consistently precedes overt compositional disruption during human neuroimmune aging remains limited. Metatranscriptomics should therefore be viewed as a promising approach for detecting early functional perturbations rather than as an established temporal marker of neuroimmune aging [162,163,169].

### 7.3. Metabolomics

Among the current multi-omics technologies, metabolomics provides one of the most direct approaches for characterizing the functional interface between the microbiome and host physiology [59,163,172]. Because metabolites represent downstream products and intermediates of microbial, host, and co-metabolic pathways, metabolomic profiles provide a closer approximation of ongoing biochemical activity than taxonomic or genomic information alone [59,172,173]. Consequently, metabolite profiles can serve as informative indicators of functional host–microbiome interactions, although source attribution and causal interpretation often require isotope tracing, microbial culture, genetic perturbation, or integrated multi-omics analyses [59,162,172].

Advances in mass spectrometry and nuclear magnetic resonance spectroscopy have enabled increasingly detailed characterization of circulating, intestinal, cerebrospinal-fluid, and tissue-specific metabolite profiles [59,172]. These approaches have identified diverse microbiome-associated molecules involved in immune and neural regulation, including short-chain fatty acids, indole derivatives, secondary bile acids, kynurenine-pathway metabolites, polyamines, and molecular cargo transported by microbial extracellular vesicles [18,28,31,52,59,68,69,70,71,173].

Metabolomic approaches have therefore shifted part of the focus of microbiome research from microbial identity toward microbial biochemical activity and inter-organ signaling [17,31,59,163]. This transition is particularly relevant to aging and neurodegenerative disorders, in which alterations in metabolic networks may occur during prodromal or preclinical stages, although their temporal relationship to disease initiation remains incompletely defined [16,46,52,118,131,174]. Experimental evidence indicates that the reduced availability of selected beneficial microbial metabolites, together with increased exposure to pro-inflammatory or neuroactive compounds, can influence microglial function and age-associated neurological vulnerability [31,51,91,124,174]. However, these effects are metabolite-, concentration-, tissue-, and context-dependent and should not be interpreted as a universal protective role for butyrate or a uniformly pathogenic role for other microbial products [31,51,124].

Metabolomics is therefore increasingly regarded as a key technology for identifying mechanistically informative candidate biomarkers and signaling pathways associated with neuroimmune aging [46,59,162,172,173,174].

### 7.4. Single-Cell RNA Sequencing

A major limitation of conventional bulk transcriptomic approaches is that averaged gene-expression measurements can obscure transcriptionally distinct cell populations and cellular states [164,165,175]. Single-cell RNA sequencing (scRNA-Seq) addresses this limitation by enabling the transcriptomic profiling of individual cells or nuclei [164,165].

Application of scRNA-Seq has transformed neuroimmune research by revealing previously unrecognized cellular subpopulations, activation trajectories, and disease-associated states [117,164,175,176,177]. In particular, single-cell studies have demonstrated that microglia do not constitute a uniform population but occupy multiple transcriptional and functional states shaped by age, brain region, tissue environment, injury, and neurodegenerative pathology [117,175,176,177]. These states include homeostatic, proliferative, interferon-responsive, lipid-associated, injury-responsive, and disease-associated transcriptional programs, whose biological effects depend on disease stage and tissue context [117,175,176,177].

Studies of aging and neurodegeneration have identified microglial transcriptional programs associated with altered inflammatory signaling, lipid and energy metabolism, phagocytosis, lysosomal function, interferon responses, and cellular stress [12,13,109,117,175,176,177]. Some of these pathways overlap with mechanisms modulated experimentally by microbiota-derived metabolites and inflammatory signals, including SCFA signaling, lipopolysaccharide-responsive pathways, and NLRP3 inflammasome activation [31,51,91,110,111,112,113]. These convergent pathways are consistent with a functional gut–immune–brain axis, but direct linkage between a specific microbial signal and a defined human microglial state generally requires complementary experimental validation [91,117,162].

Single-cell approaches have also enabled the detailed characterization of immune and stromal cell populations in the meninges, choroid plexus, perivascular spaces, and peripheral circulation [178,179]. These studies have revealed tissue-specific border-associated macrophages, lymphocyte populations, endothelial cells, fibroblasts, and choroid-plexus immune programs that participate in CNS immune surveillance and undergo age- or disease-associated remodeling [178,179,180]. Integrating these cellular atlases with microbiome, metabolomic, and longitudinal immune datasets may help determine how peripheral microbial signals influence brain-border immunity during aging [162,164,165,178,179,180].

### 7.5. Spatial Transcriptomics

Although single-cell technologies provide extraordinary cellular resolution, tissue dissociation commonly results in the loss of information regarding native tissue architecture, cellular neighborhoods, and anatomical localization [164,165,181]. Spatial transcriptomics addresses this limitation by combining gene-expression profiling with spatial localization within intact or minimally disrupted tissue sections [165,181].

This rapidly evolving technology is particularly relevant to neurodegenerative research because neuroinflammatory responses are spatially organized and depend on local cellular neighborhoods [117,177,181,182,183]. Microglia, astrocytes, endothelial cells, neurons, perivascular macrophages, and infiltrating immune cells interact within specialized anatomical and pathological niches that cannot be fully reconstructed from dissociated-cell datasets alone [165,177,178,179,181,182,183].

Spatial transcriptomic studies have identified localized transcriptional and cellular responses surrounding amyloid-β plaques, including plaque-associated microglial and astrocytic programs and altered intercellular signaling within the plaque niche [182,183]. In human Alzheimer’s disease tissue, spatially resolved expression patterns associated with amyloid-β and tau burden have also been linked to regional vulnerability and cognitive dysfunction [184]. These findings provide insight into how inflammatory, metabolic, and degenerative processes are distributed across diseased brain tissue.

Future integration of spatial transcriptomics with microbiome, metabolomic, immune, and vascular datasets may help map associations between circulating or microbiota-derived signals and specific neuroanatomical regions or cellular niches involved in aging and neurodegeneration [162,165,172,181].

Although each omics technology provides valuable information individually, their greatest potential lies in integrated analysis [162,163,164,165,166]. Metagenomics identifies microbial genetic and functional potential, metatranscriptomics captures actively transcribed microbial programs, and metabolomics reflects downstream biochemical activity, whereas single-cell and spatial transcriptomics reveal how host immune and neural cell states are distributed across cellular and anatomical contexts [59,162,163,164,165,166,167,168,169,170,172,177,181]. Together, these complementary approaches provide a systems-level framework for reconstructing dynamic gut–immune–brain interactions during aging [162,166].

Artificial intelligence and machine-learning methods are increasingly being applied to integrate high-dimensional multi-omics and clinical datasets, facilitating the identification of nonlinear molecular interactions, latent biological patterns, patient subgroups, and candidate predictive biomarker signatures that may remain undetected using conventional univariate approaches [162,166,185].

The next stage of microbiome research is likely to extend beyond isolated biomarkers toward network medicine, in which microbial, metabolic, immune, vascular, and neural components are modeled as interacting biological systems rather than independent variables [162,186]. Network-based approaches may help identify disease modules, critical signaling hubs, and cross-system pathways that contribute to neuroimmune resilience and may partly explain why apparently similar microbial alterations are associated with heterogeneous clinical phenotypes across individuals [26,115,162,186].

Integration of longitudinal multi-omics data with clinical, imaging, physiological, environmental, and lifestyle information may ultimately support the development of medical digital twins—patient-specific computational models that are dynamically updated to simulate disease trajectories and evaluate potential responses to interventions [166,187,188]. Although their use in microbiome-targeted neurodegenerative medicine remains largely conceptual, digital twins represent a promising future direction for systems biology and precision medicine [187,188].

Recent advances in biomedical foundation models may further enhance the analysis of complex host–microbiome interactions. Unlike conventional models developed for a single task or modality, foundation models are pretrained on large and heterogeneous datasets and can subsequently be adapted to multiple downstream biomedical tasks [189,190]. Future multimodal models may integrate genomic, transcriptomic, metabolomic, imaging, electronic health record, and environmental information to identify cross-modal biological relationships and generate testable hypotheses across multiple disease contexts [185,189,190].

Equally important, future computational models should prioritize interpretability, transparency, calibration, and external validation rather than predictive performance alone [191]. Post hoc explainability methods such as SHapley Additive exPlanations (SHAP) and Local Interpretable Model-agnostic Explanations (LIME) can estimate how individual microbial, metabolic, immune, vascular, or clinical variables contribute to model predictions [192,193]. However, these explanations are model-dependent, may be unstable in the presence of correlated variables, and should not be interpreted as evidence of biological causality. Explainability should therefore be combined with biological validation, uncertainty quantification, and prospective clinical evaluation [191,192,193]. Such transparency may support the clinical evaluation and regulatory assessment of AI-assisted precision microbiome approaches, although explainability alone is not sufficient for clinical acceptance.

Despite these advances, the integration of multi-omics datasets remains methodologically challenging because differences in cohort composition, biospecimen collection, sampling time, storage conditions, analytical platforms, batch effects, feature dimensionality, missing data, bioinformatic pipelines, and data harmonization can limit reproducibility and generalizability across independent studies [162,163,164,165,166,172]. Standardized sampling protocols, transparent reporting, independent external validation, and cross-cohort replication will therefore be essential before multi-omics models can be translated into clinically actionable tools [162,163,166].

### 7.6. Moving Beyond Association Studies

Despite remarkable progress, much of the current microbiome literature remains dominated by associative findings [16,19,118,125,126,131,142,156]. Demonstrating that microbial alterations coexist with neurodegenerative disease is insufficient to establish causality because observed differences may reflect confounding, reverse causation, medication exposure, dietary changes, altered intestinal transit, or consequences of the disease itself [67,156,157,194]. Future progress will therefore depend on identifying specific microbial genes, pathways, metabolites, and signaling mechanisms that directly influence neuroimmune function and determining whether their targeted manipulation modifies relevant biological or clinical disease trajectories [59,91,118,131,162,163,174].

Several complementary strategies are being used to strengthen causal inference. These include longitudinal cohort studies, germ-free and human microbiota-associated animal models, fecal microbiota transplantation experiments, targeted supplementation or depletion of microbial metabolites, Mendelian randomization analyses, and integrated systems-biology approaches [78,107,121,122,123,124,133,141,156,157,162,163,166,174,194]. Additional analytical approaches, including causal mediation analysis, Bayesian network inference, and longitudinal trajectory or time-series modeling, may further strengthen causal inference by evaluating temporal ordering, potential mediating pathways, and directional relationships among microbial, metabolic, immune, and clinical variables [162,166]. These approaches may help distinguish putative upstream microbial drivers from downstream disease-associated alterations.

Perhaps the most important conceptual shift in the field is the transition from predominantly taxonomic microbiome research toward functional microbiome biology [26,55,162,163,167]. Rather than asking only which microorganisms are present, investigators are increasingly examining which microbial genes and pathways are active, which metabolites are produced, how these signals interact with host immune and metabolic networks, and which molecular mechanisms influence disease susceptibility [17,28,31,52,59,91,162,163,168,169,170,171,172,173]. Rather than representing isolated biological layers, microbial, immune, metabolic, vascular, and neural datasets can be integrated into network-based models designed to identify functional interactions and candidate regulatory hubs associated with neuroimmune resilience across aging [115,162,166,186].

Viewed through the GIBRA framework, integrated multi-omics approaches have the potential to transform microbiome research from the descriptive characterization of microbial communities toward the systems-level identification of functional signaling networks associated with neuroimmune resilience [162,163,166]. By linking microbial genetic potential, transcriptional activity, metabolite production, immune remodeling, and tissue-specific cellular responses, these technologies may facilitate candidate biomarker discovery, strengthen causal inference, and support the development of precision microbiome-based interventions for healthy brain aging [59,162,163,164,165,166,168,169,170,171,172,173,174,175,176,177,178,179]. However, multi-omics integration alone cannot establish causality. Candidate pathways identified computationally must ultimately be validated through longitudinal replication, experimental perturbation, targeted mechanistic studies, and appropriately designed clinical interventions [156,162,163,166].

## 8. Translational Roadmap: Restoring Neuroimmune Resilience Through the Gut

The growing recognition of the gut–immune–brain axis as a dynamic systems network has stimulated considerable interest in microbiome-targeted strategies for preserving neuroimmune resilience during aging [16,19,20,44,54,91,131]. However, the translation of these concepts into clinical practice requires moving beyond the assumption that the modification of microbial composition alone will be sufficient to influence brain aging or neurodegenerative risk [26,53,54,131,156,162,195]. Within the Gut–Immune–Brain Resilience Axis (GIBRA), therapeutic interventions should instead be understood according to the biological functions they are intended to restore, including microbial and host–microbial signaling, epithelial and vascular barrier integrity, immune homeostasis, metabolic flexibility, and neuroglial regulation [28,31,43,59,75,76,77,78,81,84,86,91,102,105,106,109,173].

Importantly, these interventions should not be regarded as independent or mutually exclusive therapeutic options. Precision nutrition, psychobiotics, postbiotics, barrier-supportive strategies, immune-modulating approaches, and ecosystem-level interventions act at different but interconnected levels of the same biological network [16,19,28,31,42,54,59,75,76,77,78,91,105,106]. Their clinical value is therefore likely to depend on appropriate patient selection, mechanistic targeting, standardized and biologically characterized formulations, and longitudinal monitoring rather than on the identification of a single universally effective microbiome therapy [27,53,67,73,156,162,195,196].

The goal of such interventions is unlikely to be the reversal of biological aging itself. A more realistic translational objective is to preserve functional resilience, delay the emergence of neuroimmune dysfunction, reduce susceptibility to secondary inflammatory insults, and potentially slow or modify trajectories of cognitive decline in biologically selected individuals [8,9,10,11,14,44,53,54,108,114,115,151,152,153,196].

### 8.1. Restoring Microbial Signaling

A central principle of the GIBRA framework is that the preservation or restoration of microbial function may be more biologically informative than the reconstruction of a predefined taxonomic composition [26,55,59,162,163]. Microorganisms influence the host through diverse bioactive products and molecular signals, including short-chain fatty acids, tryptophan-derived metabolites, secondary bile acids, polyamines, microbial structural components, and extracellular vesicles [18,28,31,52,59,68,69,70,71,173]. Accordingly, interventions aimed at restoring beneficial microbial and host–microbial signaling may provide a more mechanistically tractable strategy than attempts to normalize individual bacterial taxa [17,26,54,59,162,163,195].

#### 8.1.1. Precision Nutrition

One of the most powerful and modifiable determinants of gut microbial composition and metabolic activity is precision nutrition [42,54,66,196]. Nutritional interventions influence substrate availability, microbial community structure, metabolite production, intestinal barrier function, and systemic inflammatory tone [31,42,43,47,54,66,75,76,77,78]. From a GIBRA perspective, the principal therapeutic objective of precision nutrition is not merely to alter microbiome composition, but to promote beneficial metabolic outputs and reinforce host–microbiome communication [31,42,54,59,66,173].

Fermentable dietary fiber is particularly relevant because it provides a major substrate for microbial fermentation and short-chain fatty acid production [31,32,42]. Higher intake of fermentable fiber and adherence to Mediterranean-style dietary patterns have been associated with the enrichment of selected metabolite-producing microorganisms, altered microbial metabolic output, improved metabolic homeostasis, and lower systemic inflammatory burden [42,47,54,66]. However, interindividual responses vary substantially according to baseline microbiome composition, habitual diet, medication exposure, metabolic phenotype, and host characteristics [27,42,54,67,73,196]. These differences support the development of biomarker-informed and microbiome-aware nutritional strategies rather than universal dietary prescriptions [162,196].

Polyphenols may also influence neuroimmune resilience through direct host effects and microbiome-mediated biotransformation [42,54,59,66,172]. Following intestinal and microbial metabolism, polyphenol-derived compounds can modulate redox-sensitive pathways, inflammatory signaling, endothelial physiology, and cellular metabolic responses [42,59,66,109,172,173]. Experimental findings involving flavonoids, catechins, and resveratrol are promising, but evidence for clinically meaningful and reproducible neuroprotective effects in humans remains inconclusive. Polyphenols should therefore currently be regarded as supportive modulators of dietary, microbial, and host signaling rather than as established neuroprotective therapies.

#### 8.1.2. Psychobiotics

Psychobiotics comprise selected probiotic strains—and, under broader contemporary usage, other microbiome-directed interventions—proposed to influence psychological or neural function through immune, endocrine, metabolic, and neural pathways [16,19,197]. Experimental and early clinical studies suggest that selected Lactobacillaceae and Bifidobacterium strains may modulate inflammatory signaling, hypothalamic–pituitary–adrenal axis activity, microbial metabolite production, neurotransmitter-related pathways, and neural or microglial responses [16,19,91,131,197].

Psychobiotic and probiotic effects are strain-specific and cannot be generalized to all organisms belonging to the same genus or species [197]. Clinical studies vary substantially in strain identity, formulation, dose, treatment duration, target population, concomitant therapy, and outcome assessment, which limits reproducibility and a direct comparison across studies [16,19,131,197]. In older populations, psychobiotics may therefore be more realistically conceptualized as candidate modulators of neuroimmune signaling and stress responsiveness than as established cognitive enhancers.

#### 8.1.3. Postbiotics

Postbiotics represent a potentially attractive strategy because they deliver preparations of inanimate microorganisms and/or their components without requiring administration or colonization by live microorganisms [198]. Depending on the formulation, postbiotics may contain intact inanimate microbial cells, cell fragments, and structural components, together with metabolites or other bioactive molecules retained within the characterized preparation [198]. Purified microbial metabolites administered independently of inanimate microbial biomass are better classified as microbial-derived bioactive compounds rather than postbiotics under the ISAPP consensus definition [198].

Potential biological targets of postbiotic preparations include immune regulation, redox homeostasis, mitochondrial function, epithelial integrity, endothelial stability, and neuroglial signaling [18,31,47,51,59,77,91,109,198]. Microbial-derived mediators relevant to neuroimmune aging include short-chain fatty acids, indole derivatives, secondary bile acids, and extracellular-vesicle cargo [18,28,31,51,52,59,68,69,70,71,173]. However, purified metabolites or isolated vesicles should not automatically be classified as postbiotics unless they are components of a qualifying preparation of inanimate microorganisms [198]. Potential translational advantages of well-characterized postbiotic preparations include improved stability, reduced risks associated with microbial viability or translocation, greater manufacturing control, and the possibility of standardized dosing and mechanistic characterization [195,198].

However, much of the evidence remains preclinical, and the postbiotic field continues to face limitations related to terminology, product characterization, validated potency assays, manufacturing consistency, dose selection, and robust human efficacy data [195,198]. Future development should therefore follow Quality-by-Design principles, with predefined critical quality attributes, standardized and reproducible manufacturing processes, clearly characterized bioactive component profiles, validated potency or functional assays, pharmacodynamic markers, and mechanism-based outcome assessment [195,198].

### 8.2. Restoring Barrier Integrity

Barrier dysfunction occupies a central position within the GIBRA framework because impairment of the intestinal epithelium and blood–brain barrier may increase the exposure of peripheral tissues and the central nervous system to microbial products, inflammatory mediators, and metabolite signals [75,76,77,78,81,84,85,86]. Interventions that preserve or restore barrier function may therefore help interrupt interconnected processes linking dysbiosis, microbial translocation, systemic inflammation, endothelial dysfunction, and neuroglial activation [43,75,78,79,80,81,82,83,84,85,86,91].

#### 8.2.1. Short-Chain Fatty Acids and Epithelial Homeostasis

Short-chain fatty acids, particularly butyrate, contribute to colonocyte energy metabolism, mucus production, tight-junction regulation, mucosal immune tolerance, and epigenetic control through G-protein-coupled receptor signaling and histone deacetylase inhibition [31,32,47,75,76,77]. Age-associated alterations in microbiome composition and substrate availability may reduce colonic butyrate production in some individuals, potentially contributing to impaired epithelial integrity and immune homeostasis [20,21,30,43,49,54]. Strategies that increase endogenous SCFA production through fermentable dietary fiber, dietary pattern modification, or functionally selected microbial interventions may reinforce epithelial resilience [31,32,42,47,54,66].

Direct supplementation with butyrate or other SCFAs has also been proposed, but its clinical translation is influenced by rapid intestinal absorption and metabolism, palatability, formulation, release profile, anatomical site of delivery, dose selection, and substantial differences among available preparations [31,199]. Future studies should therefore distinguish local luminal and epithelial effects from peripheral immunomodulatory activity and putative direct neuroimmune effects [31,51,59,85,124,199].

#### 8.2.2. Vitamin D

Vitamin D–vitamin D receptor signaling contributes to epithelial barrier maintenance, the regulation of tight-junction proteins, antimicrobial peptide production, innate and adaptive immunity, and control of intestinal inflammatory responses [75,76,77,102,105,200]. Vitamin D deficiency has been associated with alterations in gut microbial composition, increased intestinal permeability, and dysregulated inflammatory responses, although the direction and causal significance of these relationships remain incompletely defined [200]. Supplementation may therefore be most biologically plausible in individuals with confirmed deficiency or insufficiency rather than as a universal microbiome-directed intervention [200,201].

Observational studies have associated lower vitamin D status with cognitive impairment and dementia risk, but evidence that supplementation directly prevents neurodegeneration or produces clinically meaningful cognitive benefit remains inconclusive [201]. Within the GIBRA framework, vitamin D should therefore be regarded as a potentially supportive modifier of epithelial and immune resilience, particularly when deficiency coexists with metabolic, inflammatory, or malabsorptive conditions, rather than as an established neuroprotective therapy.

#### 8.2.3. Endothelial and Blood–Brain Barrier Protection

Restoration of neuroimmune resilience also requires attention to the vascular and neurovascular components of the axis [81,82,83,84,85,86,179]. Endothelial dysfunction, oxidative stress, chronic inflammatory activation, altered pericyte and astrocyte support, and degradation of the endothelial glycocalyx may increase blood–brain barrier vulnerability during aging [81,82,83,84,85,106,179]. Interventions that improve overall vascular health—including regular physical activity, cardiometabolic risk control, Mediterranean-style dietary patterns, smoking avoidance, and appropriate management of hypertension, diabetes, and dyslipidemia—may indirectly support neurovascular integrity [42,66,72,81,82,106,152]. Selected microbiome-derived metabolites may also modulate endothelial or neurovascular signaling, although their effects are metabolite-, concentration-, and context-dependent [28,31,51,59,85,86,124].

At present, evidence that microbiome-targeted interventions can restore blood–brain barrier integrity in humans remains limited, and most mechanistic support is derived from experimental models [85,86,91,118,131,156]. Future clinical studies should incorporate vascular and endothelial biomarkers, validated measures of intestinal permeability, neuroimaging indicators of blood–brain barrier function, and longitudinal neuroinflammatory outcomes rather than relying predominantly on taxonomic microbiome changes [81,84,85,162,179]. Such designs will be necessary to determine whether the observed microbiome changes are accompanied by the measurable restoration of barrier function and clinically relevant neurovascular benefit.

### 8.3. Reprogramming Immune Aging

Because immunosenescence and inflammaging are central and interconnected components of age-related neuroimmune decline, restoration of microbial signaling alone may be insufficient in individuals with established immune dysregulation [9,10,11,30,105,106,114,202]. A major future direction is therefore the development of interventions capable of recalibrating innate and adaptive immune responses while preserving antimicrobial defense, immune surveillance, and vaccine responsiveness [10,11,102,103,105,202].

#### 8.3.1. Inflammaging

Microbiome-targeted interventions may attenuate selected contributors to chronic inflammatory tone by reducing intestinal permeability and microbial translocation, restoring beneficial metabolite production, and modulating pattern-recognition receptor, NF-κB, and inflammasome signaling [31,32,43,50,54,59,75,78,79,80,91,110,112]. However, systemic inflammation in older adults is heterogeneous and multifactorial and can additionally arise from cellular senescence and the senescence-associated secretory phenotype, mitochondrial and metabolic dysfunction, adipose-tissue inflammation, chronic infection, cardiometabolic disease, tissue injury, and medication-related effects [8,9,10,11,49,67,104,106,114,152,202].

Consequently, reduction in maladaptive inflammaging will probably require combination strategies that integrate microbiome modulation with physical activity, nutritional optimization, management of obesity and metabolic disease, vascular risk reduction, and control of other persistent sources of immune activation [9,10,11,42,54,72,106,114,152,202].

#### 8.3.2. Trained Immunity

Repeated or persistent microbial and sterile inflammatory signals can induce long-lasting functional reprogramming of innate immune cells and hematopoietic progenitors through coordinated epigenetic, transcriptional, and metabolic remodeling, a process referred to as trained immunity [105,111,113,114]. Although trained immunity can enhance heterologous protection against subsequent infections, persistent or maladaptive training may contribute to exaggerated inflammatory responsiveness and chronic inflammatory disease [105,111,114].

This raises the possibility that future interventions could target not only the circulating cytokine concentrations but also the cellular and progenitor-level programs that sustain exaggerated innate immune responses [105,111,202]. At present, however, this remains predominantly a mechanistic and preclinical concept. Clinical studies are needed to determine whether dietary interventions, microbial metabolites, defined postbiotic preparations, or pharmacological strategies can safely modify maladaptive innate immune memory without compromising host defense in older adults [31,59,105,111,195,198,202].

#### 8.3.3. Immunometabolism

Immune activation is tightly coupled to cellular metabolism [109,202,203]. Many pro-inflammatory states of peripheral myeloid cells and microglia are associated with increased glycolytic flux and the remodeling of mitochondrial and tricarboxylic-acid-cycle metabolism, although the direction and magnitude of these changes depend on cell type, stimulus, tissue environment, and disease stage [109,110,112,203]. In selected pro-inflammatory myeloid-cell states, disruption of tricarboxylic-acid-cycle flux can promote the accumulation of metabolites such as succinate, the stabilization of hypoxia-inducible factor 1α, increased reactive oxygen species production, and the enhanced expression of inflammatory mediators [110,112,203].

Microbiome-derived metabolites may influence immunometabolic programs through G-protein-coupled receptors, aryl hydrocarbon receptor signaling, mitochondrial substrate availability, redox regulation, and epigenetic mechanisms such as histone deacetylase inhibition [28,31,32,52,59,68,69,70,71,109,173]. Future GIBRA-oriented interventions should therefore incorporate immunometabolic endpoints—including mitochondrial respiration, glycolytic capacity, redox status, metabolite flux, and inflammasome-related activity—rather than evaluating immune modulation solely through circulating cytokine concentrations [103,110,162,164,172,202,203]. Where feasible, these endpoints should be assessed using functional metabolic and protein-level measurements rather than inferred exclusively from transcript abundance [110,164,172,203].

### 8.4. Precision Microbiome Medicine

The marked biological and clinical heterogeneity of aging represents a major obstacle to translating microbiome research into clinical practice [20,21,22,44,45,46,48,53,54,114,115,151,202,204]. Older adults differ in microbial functional capacity, metabolite production, barrier integrity, inflammatory and vascular status, cognitive reserve, medication exposure, lifestyle, comorbidities, and neurodegenerative pathology [20,21,44,46,48,54,67,114,115,151,152,153,202,204]. Accordingly, precision microbiome medicine should prioritize biologically meaningful endotypes capable of predicting differential responses to nutritional, microbial, barrier-supportive, or immune-modulating interventions rather than assuming uniform effects across unselected populations [27,46,53,54,67,73,156,162,166,195,196,204].

#### 8.4.1. Biomarker Integration

No single biomarker can adequately capture the multidimensional biology of neuroimmune aging [9,10,20,21,46,106,114,162,205]. Precision approaches should therefore integrate microbial functional capacity and metabolites with markers of barrier integrity and microbial translocation, systemic inflammation, endothelial/BBB dysfunction, neuroglial activation, and neuroaxonal injury [31,46,52,59,75,81,82,83,84,85,86,162,163,164,165,166,172,179,204,205,206]. Multidimensional panels may better characterize biological endotypes and longitudinal trajectories than isolated markers, but their added value requires external validation and comparison with simpler clinical models [162,166,185,186,204,205].

Candidate biomarkers include SCFAs, tryptophan-derived indoles, secondary bile acids, markers of intestinal barrier dysfunction and microbial translocation such as LBP, circulating inflammatory mediators, GFAP, NfL, sTREM2, and selected endothelial/vascular biomarkers [28,31,46,52,59,75,78,79,81,82,83,84,85,106,117,128,152,172,179,205,206]. None should be considered disease-specific in isolation, because concentrations may vary with age, renal function, cardiovascular and metabolic disease, inflammation, assay platform, biological compartment, and coexisting neurological pathology [205,206].

Table 3 summarizes 18 biomarker categories spanning microbial metabolism, barrier dysfunction and translocation, systemic inflammation, neuroglial and neuroaxonal injury, and vascular dysfunction as a hypothesis-driven framework for multidimensional patient stratification.

Important methodological limitations particularly apply to barrier-related biomarkers. Commercial zonulin assays may not specifically quantify pre-haptoglobin-2, and circulating zonulin-related immunoreactivity does not consistently correspond to direct functional permeability measures [75,207]. Similarly, LBP reflects the host response to bacterial LPS exposure rather than directly measuring intestinal permeability or microbial translocation and is influenced by hepatic inflammation, infection, obesity, and metabolic disease [50,75,78,79,208]. These limitations support the interpretation of barrier, translocation, inflammatory, and clinical indicators within multidimensional panels rather than in isolation [162,166,204,205].

These limitations reinforce the need to combine barrier, microbial-translocation, inflammatory, and clinical indicators in multidimensional panels rather than interpreting individual measurements in isolation [162,166,204,205].

From a translational perspective, integration of these molecular layers could enable the definition of functional neuroimmune endotypes, moving beyond stratification based on isolated biomarker measurements [162,166,170,204,205,206]. For example, a metabolite-deficient endotype could combine reduced microbial capacity for butyrate biosynthesis with decreased SCFA availability and evidence of impaired intestinal barrier function [31,46,59,75,78,172]; a tryptophan–inflammatory endotype could integrate altered microbial indole pathways, an increased kynurenine-to-tryptophan ratio, and elevated systemic inflammatory mediators [52,59,68,69,70,71,106,173]; and a barrier–translocation endotype could combine markers of intestinal permeability or epithelial injury with LBP and inflammatory activation [50,75,78,79,207,208]. At the neuroimmune level, these peripheral signatures could be integrated with GFAP, NfL, sTREM2, vascular or endothelial biomarkers, neuroimaging, and cognitive phenotypes to identify individuals with different patterns of neuroglial activation and neuroaxonal vulnerability [81,82,83,84,86,117,128,179,205,206,209,210,211,212,213]. Such composite signatures remain hypothesis-generating and require prospective validation, but they illustrate how multi-omics integration could move patient stratification from isolated biomarker abnormalities toward biologically coherent pathway-based endotypes capable of informing mechanism-based intervention selection [162,166,185,204,205,206].

#### 8.4.2. Multi-Omics Integration

Metagenomics, metatranscriptomics, metabolomics, proteomics, single-cell sequencing, and spatially resolved methods provide complementary information about microbial functional potential, active gene expression, metabolite production, host-cell states, and tissue-specific biological responses [162,163,164,165,167,168,170,172,177,178,179,180,181,182,183,184]. Integration of these data layers may enable the identification of functional microbiome endotypes, host–microbial interaction patterns, and mechanistically relevant signaling networks that cannot be resolved from taxonomic profiles alone [26,55,59,162,163,166,170,172,173,186].

Recent studies illustrate how multi-omics integration can move beyond a parallel description of individual molecular layers toward reconstruction of coordinated microbial–host interaction networks. In experimental aging, the integration of metagenomics, metabolomics, host transcriptomics, and metabolic modeling identified age-associated deterioration of host–microbiome metabolic exchange, linking altered microbial functions, including butyrate- and bile-acid-related metabolism, with host transcriptional and metabolic responses across multiple tissues [58]. Complementary human evidence from older adults showed that integrated gut metagenomic and plasma metabolomic profiling identified microbial–metabolic signatures associated with frailty severity and mortality, thereby linking microbial ecosystem features with systemic biochemical and clinical phenotypes [36]. In Parkinson’s disease, integration of metagenomics, metatranscriptomics, metaproteomics, and meta-metabolomics further demonstrated that functional alterations may emerge at transcriptional and metabolic levels even in the absence of corresponding changes in microbial gene abundance, while cross-omics network analysis connected specific microbial activities with altered metabolite profiles [215]. Similarly, across the Alzheimer’s disease continuum, integration of gut microbial profiles, fecal metabolomics, multimodal brain MRI, and cognitive measures identified candidate microbiota–metabolite–brain–cognition pathways, illustrating how peripheral microbial and metabolic alterations can be linked to structural and functional brain phenotypes [216]. Collectively, these studies define a layered analytical logic in which metagenomic data characterize microbial functional potential, transcriptional and proteomic layers identify active biological programs, metabolomics captures downstream biochemical output, and host molecular, imaging, and clinical phenotypes define the corresponding biological response. Cross-layer integration can therefore prioritize microbial–metabolite–host interaction modules for subsequent mechanistic validation rather than relying on associations within any single omics layer. Nevertheless, fully integrated longitudinal datasets simultaneously combining repeated microbiome functional profiling with host immune, neuroinflammatory, and CNS phenotyping remain limited, particularly in human aging and neurodegenerative disease.

However, multi-omics approaches remain constrained by high cost, limited sample size, batch effects, pre-analytical variability, missing data, differences in analytical and computational pipelines, and incomplete standardization across cohorts and platforms [162,163,164,165,166,168,169,170,171,172]. Translation into clinical practice will require reproducible and analytically validated assays, harmonized sampling and processing protocols, transparent computational workflows, external validation in independent and demographically diverse populations, and outputs that can be interpreted in relation to clinically relevant biological processes [162,166,170,172,204,217,218].

#### 8.4.3. Artificial Intelligence and Predictive Modeling

Artificial intelligence and machine-learning methods may facilitate the integration of high-dimensional microbial, immunological, metabolic, imaging, and clinical datasets [162,166,185,186,187,188,189,190]. Potential applications include endotype discovery, multidimensional biomarker identification, treatment-response prediction, detection of nonlinear interactions, and longitudinal risk stratification [185,186,187,188,189,190,217,218].

In practical terms, supervised machine-learning approaches such as random forests, gradient-boosting algorithms (e.g., XGBoost), support vector machines (SVMs), and, where sufficiently large datasets are available, neural networks could integrate microbial functional features with metabolite profiles, inflammatory and barrier biomarkers, neuroglial or vascular markers, neuroimaging, and clinical variables to derive multivariable signatures for patient stratification or treatment-response prediction. Such models could test whether combinations of reduced SCFA-related functional capacity, altered tryptophan metabolism, increased LBP and inflammatory signaling, and elevated GFAP or NfL distinguish biologically defined endotypes more effectively than individual biomarkers or taxonomic features alone. Their clinical value, however, would require a comparison with simpler reference models, rigorous calibration, external validation, and prospective evidence that model-informed stratification improves clinically relevant decision-making [162,166,185,186,187,188,189,190,191,192,193,217,218].

Apparent performance in development datasets is insufficient for clinical implementation [191,217,218]. Predictive models require rigorous internal and external validation, assessment of calibration, discrimination and clinical utility, and where appropriate, prospective real-world testing [217,218]. Model development should also address data leakage, overfitting, class imbalance, missingness, subgroup performance, and algorithmic bias [217,218]. Biological plausibility and interpretability should be considered alongside statistical performance, particularly when models are intended to define endotypes or guide therapeutic decisions [186,191,192,193,218].

Explainable and transparent models are therefore particularly important for patient stratification and therapeutic decision support [191,192,193,218]. However, explainability methods describe how a model generates predictions and should not be interpreted as evidence of biological causality [191,192,193].

### 8.5. Clinical Implementation

Successful clinical translation of the Gut–Immune–Brain Resilience Axis (GIBRA) will require reproducible, biomarker-guided workflows based on functional rather than purely taxonomic endotyping [162,166,195,204,205,217,218]. Potential endotypes may include metabolite-deficient, barrier dysfunction-associated, endotoxemia-dominant, inflammaging-dominant, neuroglial activation-associated, vascular dysfunction-associated, or multidomain phenotypes [26,31,46,50,51,52,53,54,59,75,78,79,85,106,152,162,166,195,196,204,206,207,208,209,210,211,212,213,214]. Such stratification could support patient selection, intervention matching, and treatment monitoring, but remains hypothesis-generating and requires prospective validation before clinical implementation [26,55,162,163,166,172,195,196,204,205,217,218].

#### 8.5.1. Patient Stratification

Patient selection should be aligned with the biological mechanism and therapeutic target [195,196,204]. For example, fermentable fiber, prebiotic, or defined postbiotic approaches may be most relevant in individuals with reduced production or systemic availability of beneficial metabolites such as SCFAs, whereas barrier-supportive interventions may be more appropriate when evidence indicates intestinal barrier dysfunction or microbial translocation [31,42,54,66,75,78,197,198,199,204]. However, validated treatment-selection thresholds are currently lacking; mechanism-based matching should therefore be tested prospectively rather than applied as routine clinical guidance [75,204,205,207,208].

Disease stage may also modify therapeutic responsiveness [6,7,81,84,128,130,131,205]. Interventions aimed at prevention or disease modification may have greater potential during preclinical or prodromal stages, before extensive neuronal loss, although the optimal therapeutic window is likely to vary by disease, intervention, and biological target and requires direct testing [6,7,81,84,130,131,205].

#### 8.5.2. Clinical Trial Design

Future trials should replace biologically heterogeneous populations and poorly characterized microbiome interventions with biomarker-informed, mechanism-driven designs using standardized therapeutic products [156,157,195,197,198,199,204]. Studies should incorporate biologically justified dosing and duration; microbiome and metabolite profiling; assessment of barrier integrity, systemic inflammation, neuroglial activation, and neuroaxonal injury; and prespecified procedures for biospecimen collection, processing, analytical platforms, quality control, and statistical interpretation [31,75,162,166,172,195,197,198,199,204,205,206,207,208,217]. Clinical efficacy should be assessed using standardized cognitive, functional, and patient-centered outcomes with longitudinal follow-up, adherence assessment, medication and dietary documentation, and safety monitoring [33,67,130,131,204]. Importantly, trials should distinguish target engagement, biological response, and clinical efficacy, rather than equating microbiome compositional change with therapeutic benefit [26,54,55,162,167,172,204].

Adaptive and biomarker-enriched designs may identify biologically defined responders and reduce treatment-effect dilution caused by heterogeneity [166,196,204,205,217]. Their validity will require prespecified biomarker hypotheses, analytically validated assays, multiplicity control, protection against overfitting, and independent confirmation of treatment–biomarker interactions [162,166,204,205,217]. Within GIBRA, these approaches could facilitate progression from empirical microbiome interventions toward precision strategies matched to biological endotypes [162,166,195,196,204,205].

The quantity, methodological maturity, and clinical reproducibility of evidence differ substantially across microbiome-targeted strategies [22,54,118,125,126,131,143,195,197,204]. Their current translational status is summarized in Table 4.

FMT illustrates both the mechanistic potential and translational limitations of ecosystem-level microbiome manipulation. In animal models, heterochronic microbiota transfer can modify age-associated barrier dysfunction, systemic inflammation, neuroinflammatory signaling, and selected behavioral or cognitive phenotypes, whereas aged microbiota may reproduce or accelerate some age-related abnormalities [123,141,227]. However, these findings remain preclinical and cannot be directly extrapolated to humans [156,157,227]. Randomized trials in Parkinson’s disease have yielded inconsistent results, while donor selection, product characterization, engraftment, durability, long-term safety, procedural standardization, and regulatory oversight remain unresolved [131,156,166,184,185,204,224,225]. FMT should therefore remain an experimental ecosystem-level intervention for neurodegenerative indications [195,204,224,225].

### 8.6. From Therapeutic Concept to Precision Clinical Pathway

Translation of GIBRA-based interventions into clinical practice will require a structured precision-medicine pathway integrating functional endotyping, biomarker-guided patient selection, mechanism-matched and compositionally defined interventions, and longitudinal assessment of biological and clinical responses [162,166,195,204,205,217,218]. Such a framework should distinguish target engagement, biological response, and clinical efficacy and incorporate predefined criteria for treatment adaptation or discontinuation [166,195,204,205,217].

Rather than a single therapeutic modality, precision microbiome medicine may evolve into an integrated platform combining personalized nutrition, defined microbial or metabolite-based therapeutics, multidomain biomarkers, multi-omics, and interpretable computational decision support [162,163,164,165,166,172,185,186,187,188,189,190,191,192,193,194,195,196,197,198,204,205,206,217,218]. Integration of microbial functional capacity with barrier, immune-inflammatory, neuroglial and neuroaxonal biomarkers, disease stage, comorbidities, medications, diet, and longitudinal outcomes could support individualized treatment selection [46,67,75,162,166,172,204,205,206,207,208,209,210,211,212,213,214,215,216,217,218]. However, this model remains aspirational and will require validated biomarkers, standardized sampling and therapeutic products, externally validated algorithms, regulatory and cost-effectiveness assessment, and the prospective demonstration of benefit over simpler non-stratified approaches [162,166,195,204,205,217,218].

Overall, the mechanistic plausibility of microbiome-targeted interventions currently exceeds the strength and reproducibility of their clinical validation [22,54,118,125,126,131,143,195,197,204,221,222,223,224,225]. Their relative evidence maturity and potential clinical positioning are summarized in Table 5.

Viewed comparatively, microbiome-targeted interventions occupy different positions along the translational continuum and should not be assigned equivalent clinical priority. Mediterranean/MIND and fiber-rich dietary approaches currently have the broadest applicability as low-risk preventive or supportive strategies, although evidence for microbiome-mediated neurodegenerative disease modification remains insufficient [42,54,66,219,220]. Probiotics and psychobiotics remain adjunctive and limited by strain-, formulation-, dose-, and population-specific heterogeneity [143,197,221], whereas postbiotics offer greater compositional control but remain predominantly preclinical or early translational for neurodegenerative indications [195,198]. FMT provides ecosystem-level microbiome manipulation but carries substantial donor, engraftment, safety, standardization, procedural, and regulatory challenges, with inconsistent clinical evidence supporting its continued experimental status [131,195,204,224,225]. Overall, the current evidence therefore favors a translational hierarchy from broadly applicable dietary strategies toward increasingly targeted but less clinically validated interventions, guided by biological endotype, disease stage, safety, and target engagement rather than a universal treatment sequence [162,166,195,204,205,217,218].

Collectively, microbiome-targeted interventions remain promising complementary strategies for preserving neuroimmune resilience [16,19,22,54,118,131,143,195,197,204], but cannot currently be considered established disease-modifying treatments for Alzheimer’s disease, Parkinson’s disease, or age-related cognitive decline [118,125,126,131,143,204,219,221,222,223,224,225,228] (Table 6). Clinical translation will require stronger causal evidence, reproducible and compositionally defined therapeutic products, validated multidomain biomarkers, biologically informed patient stratification, and prospective demonstration of clinically meaningful benefit [162,166,195,204,205,217,218].

### 8.7. Limitations

Although this review integrates current evidence into the proposed Gut–Immune–Brain Resilience Axis (GIBRA) framework, several limitations should be acknowledged. In addition, because this work was designed as a structured narrative rather than a systematic review, study identification and selection were not conducted within a preregistered protocol or formal study-level risk-of-bias framework; consequently, selective inclusion and interpretative subjectivity cannot be fully excluded despite the structured search strategy and predefined thematic scope. First, much of the available evidence remains associative rather than causal, and many reported microbiome signatures have not yet been consistently reproduced across independent cohorts owing to differences in study populations, sequencing methodologies, and analytical pipelines.

Second, the current literature incompletely assesses and controls for major determinants of microbial composition and function, including diet, medication exposure, physical activity, intestinal transit, frailty, comorbidity burden, geography, living environment, and host metabolic status. These factors can substantially influence microbial functional capacity and metabolite availability and may therefore confound apparent associations among chronological age, microbiome alterations, and neuroimmune phenotypes. Consequently, some changes attributed to “microbiome aging” may reflect, at least in part, correlated environmental, clinical, or physiological exposures rather than chronological aging per se.

Future clinical studies should therefore prospectively collect and harmonize data on dietary intake, long-term medication exposure, recent antibiotic use, physical activity, intestinal transit, frailty, comorbidities, and relevant environmental factors, and incorporate these variables through predefined exclusion criteria, stratification, matching, or multivariable adjustment, as appropriate. Repeated sampling across geographically and environmentally diverse populations, together with external validation, will be particularly important for distinguishing reproducible age- or disease-associated microbial signatures from context-dependent effects.

Third, despite the increasing availability of longitudinal datasets, most human studies remain cross-sectional, limiting the ability to determine whether microbiome alterations precede, accompany, or result from neurodegenerative processes.

Fourth, a substantial proportion of mechanistic knowledge derives from animal models, which have been instrumental for establishing biological plausibility but cannot fully recapitulate the complexity and interindividual variability of the human microbiome or human neuroimmune aging.

Furthermore, although multidomain biomarker panels and multi-omics technologies represent promising tools for precision medicine, their analytical standardization, external validation, clinical reproducibility, and cost-effective implementation remain incomplete. Finally, GIBRA should be regarded as a conceptual and hypothesis-generating framework that integrates current mechanistic knowledge rather than a clinically validated model for patient stratification or therapeutic decision-making. Future prospective longitudinal studies, biomarker-guided clinical trials, and external validation across diverse populations will be essential to determine its clinical utility and translational value [20,22,53,54,156,157,162,163,164,165,166,170,172,204,205,207,217,218].

## 9. Future Directions

Although substantial progress has been made in understanding the relationship between the gut microbiome and neuroimmune aging, translation into clinically meaningful interventions will require a shift from descriptive associations toward mechanistic, systems-level approaches capable of resolving causal pathways linking microbial dysfunction to neuroimmune resilience and neurodegenerative vulnerability [16,19,156,162,166,204].

A major priority is the development of large-scale longitudinal human studies with repeated microbiome sampling across the aging trajectory [56,57,162,166,170,204]. Because microbial and immune profiles are temporally dynamic, cross-sectional studies provide only a static representation of this ecosystem [20,22,44,45,166,169,170]. Longitudinal integration of microbial, immunological, metabolic, cognitive, and, where feasible, neuroimaging data will be essential for identifying early biomarkers and determining whether microbiome alterations precede, accompany, or result from neurodegenerative processes [162,163,164,165,166,170,171,172,204].

Future clinical trials should likewise move from broad diagnostic categories toward mechanism-based and biomarker-informed designs [195,204]. Integrated phenotyping incorporating microbiome profiles, metabolomics, inflammatory biomarkers, neuroimaging, and clinical characteristics may identify therapeutically actionable pathways and biologically distinct neuroimmune endotypes [46,162,163,164,165,166,170,171,172,204,205]. Such stratification is particularly relevant given the substantial heterogeneity in microbial composition, immune status, metabolic function, and neuroinflammatory burden among older individuals [20,21,46,53,54]. Candidate markers—including zonulin, LBP, IL-6, TNF-α, NfL, and GFAP—may contribute to multidomain endotyping, although their clinical use will require appropriate analytical and prospective validation [75,204,205,206,207,208,209,210,217].

Multi-omics integration will be central to this transition [162,163,164,165,166,170,171,172]. Combining metagenomics, metatranscriptomics, metabolomics, single-cell and spatial transcriptomics, and systems biology may help distinguish causal microbial mechanisms from secondary disease-associated changes and connect microbial functional potential with immune, metabolic, vascular, and neurobiological phenotypes [156,162,163,164,165,166,170,171,172,186]. Rather than analyzing isolated molecular layers, future studies should therefore reconstruct interacting host–microbiome networks and their evolution across aging.

**Figure 6:**
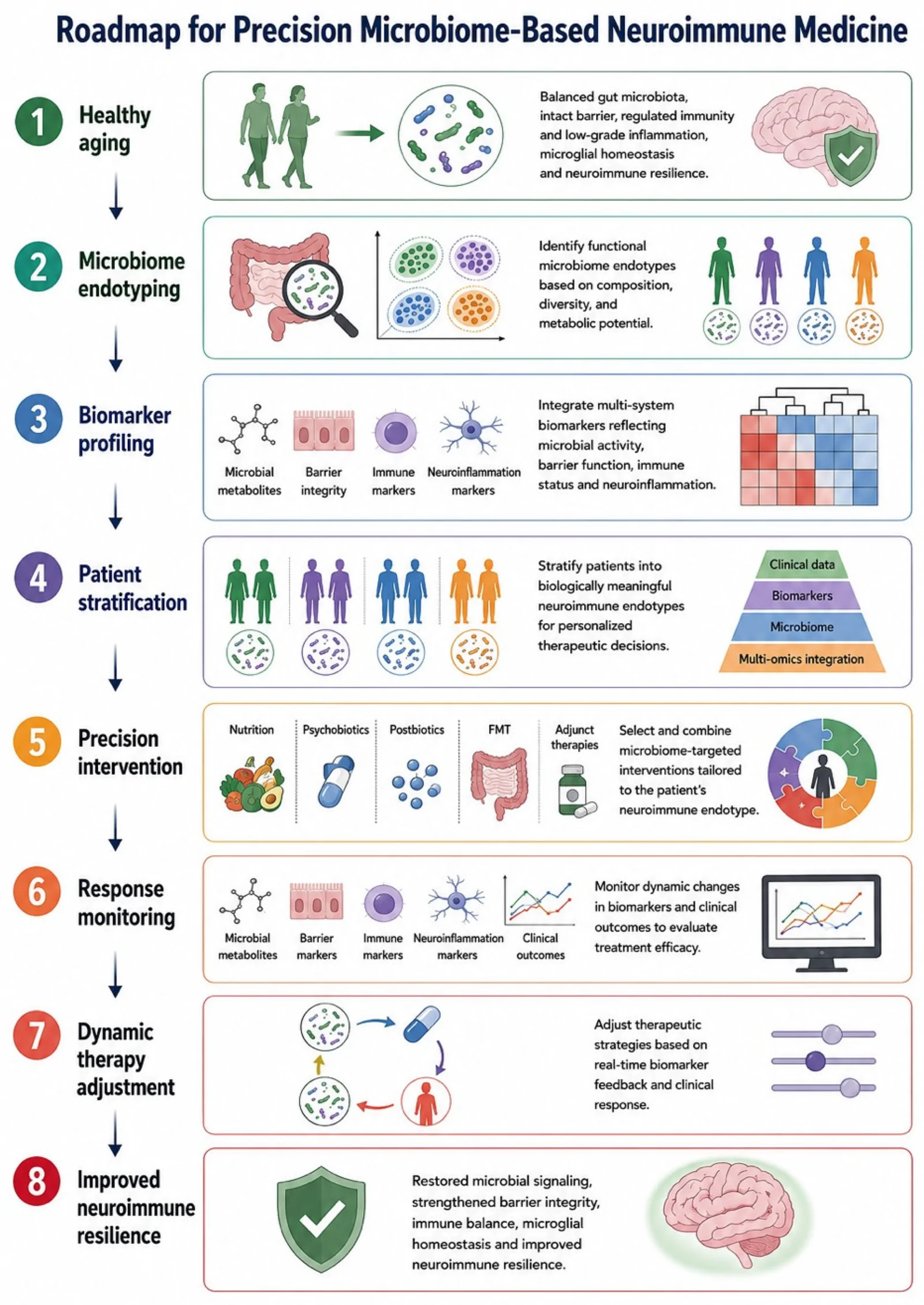
Translational roadmap for precision microbiome-based neuroimmune medicine within the Gut–Immune–Brain Resilience Axis (GIBRA). Proposed conceptual framework illustrating the progression from healthy aging toward precision microbiome-based neuroimmune medicine. The roadmap integrates functional microbiome endotyping, multidomain biomarker profiling, patient stratification, and mechanism-based therapeutic selection. Rather than relying solely on microbial taxonomy, the framework incorporates microbial metabolites, intestinal barrier integrity, systemic inflammation, neuroglial activation, neuroaxonal injury, and vascular biomarkers to define biologically meaningful neuroimmune endotypes. Longitudinal biomarker monitoring enables iterative treatment refinement aimed at restoring microbial signaling, barrier integrity, immune homeostasis, and neuroimmune resilience.

Future precision microbiome medicine is unlikely to rely on universal interventions or generic probiotic supplementation, but rather on individualized strategies matched to microbial, metabolic, immunological, and neuroinflammatory endotypes (Figure 6) [85,195,196,197,198]. Candidate approaches include targeted microbial metabolites, engineered microbial consortia, postbiotics, extracellular vesicles, and personalized nutritional interventions designed to restore specific signaling pathways [195,198]. Artificial intelligence and machine-learning approaches may assist by integrating multidimensional microbial, immune, barrier, neurobiological, imaging, and clinical data to support biomarker discovery, patient stratification, and therapeutic-target identification [185,186,189,190,191,192,193,194,204,205,206,217,218]. Digital twin technologies may eventually extend this concept by integrating longitudinal microbiome, multi-omics, neuroimaging, and clinical data to simulate individual resilience trajectories and potential preventive strategies, although these applications remain developmental [187,188,204,217,218].

Future precision microbiome medicine will increasingly require the integration of targeted interventions—including microbial metabolites, engineered microbial consortia, postbiotics, extracellular vesicles, and personalized nutrition—with biomarker-guided stratification and multidimensional data analysis [195,198]. Artificial intelligence and machine-learning approaches may support this transition by integrating microbial, metabolic, immune, barrier, neurobiological, imaging, and clinical data for biomarker discovery, patient stratification, and therapeutic-target identification [185,186,189,190,191,192,193,194,204,205,206,217,218]. Digital twin technologies may eventually extend this framework by combining longitudinal microbiome, multi-omics, neuroimaging, and clinical data to model individual resilience trajectories and potential preventive strategies, although these applications remain developmental [187,188]. Ultimately, progress will depend on integrating longitudinal human cohorts, biomarker-guided stratification, AI-assisted multi-omics, and precision microbiome interventions within a unified resilience-oriented framework [162,166,204,205,206,217,218]. GIBRA provides a conceptual basis for investigating how interactions among the microbiome, immune system, biological barriers, and brain shape individual resilience trajectories across aging [16,19,54,85,91]. Rather than focusing exclusively on established neurodegenerative disease, future research may increasingly seek to preserve neuroimmune resilience before irreversible pathology develops, shifting emphasis from disease management toward resilience-based prevention [8,10,53,54,162].

## 10. Conclusions

The gut microbiome is increasingly recognized as an active participant in the biological processes that shape brain aging [16,19,20,54]. Rather than serving merely as a biomarker of age-related decline, the aging microbiome functions as a dynamic regulator of immune homeostasis, metabolic signaling, barrier integrity, and neuroinflammatory responses.

In this review, we propose that the age-associated disruption of microbial signaling pathways represents a critical upstream event linking dysbiosis to neuroimmune aging. Loss of beneficial microbial metabolites, increased microbial translocation, chronic systemic inflammation, and immune remodeling converge on microglial priming, which may serve as a central mechanistic hub connecting peripheral aging processes with neurodegenerative vulnerability [30,31,51,78,91,106,108,116].

Viewed through this framework, immunosenescence, inflammaging, intestinal barrier dysfunction, blood–brain barrier disruption, and microglial activation should not be considered isolated phenomena. Instead, they represent interconnected components of a broader gut–immune–brain axis that progressively influences the resilience of the aging nervous system [53,54,75,81,85,86,91].

A key message emerging from the current evidence is that microbial function may be more important than microbial composition alone. Future advances will likely depend on identifying the specific microbial signaling pathways that regulate immune and neural homeostasis, rather than focusing exclusively on individual bacterial taxa. This shift from taxonomic description toward functional and mechanistic understanding represents one of the most important conceptual developments in contemporary microbiome research [26,31,52,55,59,195].

Although important questions remain regarding causality, patient heterogeneity, and therapeutic implementation, advances in multi-omics technologies, biomarker-guided stratification, and precision microbiome interventions are rapidly transforming the field. These developments support a future in which microbiome-targeted strategies may complement existing approaches to promote healthy aging and preserve brain function [162,166,185,204,205,206,217,218].

Ultimately, the aging gut microbiome should be viewed not only as a determinant of gastrointestinal health, but also as a modifiable regulator of neuroimmune resilience. Understanding and restoring microbial signaling networks may open new opportunities for preventing or delaying neurodegenerative processes and fostering healthier cognitive aging across the lifespan [31,52,54,59,91,155,195].

## Figures and Tables

**Table 1:** Representative quantitative human evidence supporting functional microbiome profiles across healthy longevity, frailty, Alzheimer’s disease, and Parkinson’s disease.

Phenotype	Representative Functional/Metabolic Evidence	Inflammatory/Barrier Evidence	Interpretation for Functional Endotyping
Healthy longevity/centenarians	In a multi-omics cohort of 425 participants including 145 centenarians, centenarians showed distinct serum metabolomic and gut/oral microbial profiles; tryptophan metabolism was enriched, and 5-methoxyindoleacetic acid (5-MIAA) emerged as a longevity-associated tryptophan metabolite linked to *Christensenellaceae R-7 group* [33]. Healthy centenarians also show age- and diet-associated remodeling of SCFA, amino acid, and phospholipid metabolism [34,35].	Qiu et al. further reported differences between healthy and frail centenarians and the experimental anti-inflammatory/senescence-modulating effects of 5-MIAA [33].	Healthy longevity appears to be associated with the preservation or remodeling of microbial metabolic function rather than a single taxonomic configuration, particularly involving the tryptophan- and SCFA-related pathways.
Frailty	In 1821 older adults, integrated metagenomics and plasma metabolomics identified 18 microbial species and 17 circulating metabolites that varied with frailty severity; a microbiome-derived frailty score predicted 2-year mortality (adjusted HR 2.86; 95% CI 1.38–5.93) [36].	Longitudinal evidence indicates that higher inflammatory burden, particularly hs-CRP, is associated with subsequent worsening of frailty over 8 years [37].	Frailty is characterized by a multidimensional microbial–metabolic–inflammatory phenotype rather than by the loss of a single microbial taxon or metabolite, supporting composite functional endotyping.
Alzheimer’s disease/AD continuum	In 287 individuals across the AD continuum, fecal propionate, isovalerate, and propionate-producing bacteria were inversely associated with amyloid positivity, and SCFA levels were associated with slower cognitive decline [38]. Plasma kynurenine-pathway profiling in AD showed significant alterations in tryptophan, xanthurenic acid, 3-hydroxyanthranilic acid, and quinolinic acid [39].	SCFA associations were linked to AD biomarker status and cognition, while kynurenine-pathway abnormalities demonstrate that host–microbial tryptophan metabolism may accompany the AD phenotype [38,39].	AD-related functional profiles may involve both impaired SCFA-associated signaling and altered tryptophan/kynurenine metabolism, but effects are heterogeneous and cannot yet define a validated AD-specific metabolic endotype.
Parkinson’s disease	In 96 PD patients and 85 controls, fecal acetate, propionate, and butyrate were lower, whereas their plasma concentrations were higher in PD. Lower fecal butyrate was associated with greater motor severity (ρ = −0.40, *p* = 0.004) [40]. In an independent cohort of 55 PD patients and 56 controls, reduced fecal SCFA concentrations were also observed [41].	Aho et al. reported increased fecal calprotectin together with reduced fecal SCFAs, indicating concurrent intestinal inflammatory and metabolic alterations. Several associations between microbiota, SCFAs, and inflammatory markers were sex-dependent, while fecal and plasma inflammatory markers were not significantly correlated [41].	PD therefore provides an example of compartment-specific metabolic alterations, as fecal and circulating SCFAs may show divergent patterns. These findings highlight the importance of considering the biological matrix and relevant clinical and host-related factors when interpreting microbiome-derived metabolite profiles.

Note: Quantitative values and statistical associations summarized in this table originate from independent human studies and should not be interpreted as direct head-to-head statistical comparisons among healthy centenarians, frail older adults, individuals with Alzheimer’s disease, and patients with Parkinson’s disease. The studies differ substantially in cohort composition, biological matrices, analytical platforms, dietary and medication exposure, intestinal transit, disease stage, and statistical adjustment. Accordingly, the table summarizes representative phenotype-associated functional signatures rather than validated cross-disease thresholds. Healthy longevity is characterized predominantly by preserved or remodeled metabolic capacity, including tryptophan- and SCFA-related pathways; frailty by integrated microbial–metabolic signatures accompanied by chronic inflammatory burden; Alzheimer’s disease by heterogeneous SCFA and tryptophan/kynurenine abnormalities associated with amyloid status and cognitive trajectories; and Parkinson’s disease by particularly pronounced compartment-dependent SCFA abnormalities together with intestinal inflammatory features. These patterns support the biological plausibility of functional microbiome endotypes but also demonstrate why harmonized prospective cohorts using standardized sampling and multi-omics measurements are required before formal quantitative cross-phenotype classification can be established.

**Table 2:** Current evidence supporting the gut–immune–brain axis in neurodegeneration: contributions and limitations across human and experimental studies.

Evidence Level	Major Contributions	Principal Limitations
Human observational studies (cross-sectional, prospective, and longitudinal cohorts)	Identify clinically relevant associations between microbial taxonomic and functional profiles, metabolite-related pathways, barrier and inflammatory markers, and cognitive phenotypes; emerging prospective and longitudinal studies provide information on temporal associations with cognitive trajectories	Predominantly observational evidence; limited repeated functional microbiome and metabolomic sampling; high interindividual heterogeneity; residual confounding by diet, medications, lifestyle, frailty, comorbidities, intestinal transit, and other host factors; limited ability to establish temporal directionality or causality
Conventional animal models	Provide mechanistic evidence linking microbiome perturbation and microbial metabolites with intestinal and blood–brain barrier dysfunction, systemic immune activation, microglial responses, neuroinflammation, and behavioral phenotypes	Species-specific differences in microbiome composition, immune and neurovascular biology, lifespan, diet, and environmental exposures limit direct translation to human neuroimmune aging
Germ-free and microbiota-depletion models	Demonstrate the requirement of microbiota-derived signals for normal immune and microglial maturation and enable investigation of the effects of microbial depletion or absence on host physiology	Lifelong absence of microbial colonization profoundly alters immune and neurodevelopmental maturation; antibiotic-mediated depletion has off-target and context-dependent effects; neither model fully recapitulates naturally occurring age-associated dysbiosis
Fecal microbiota transplantation (FMT) models	Test whether donor-associated microbial communities can transfer components of metabolic, inflammatory, neuroimmune, or behavioral phenotypes and provide stronger evidence for microbiome-dependent effects than observational associations alone	Variable donor characteristics, recipient background and age, engraftment efficiency, housing and dietary conditions, and experimental protocols; transferred phenotypes may not identify the specific microbial taxa, functions, or metabolites responsible
In vitro and ex vivo models	Define specific molecular and cellular mechanisms, including TLR4/NF-κB signaling, HDAC inhibition, NLRP3 activation, cytokine responses, epithelial and endothelial barrier regulation, and metabolite–host interactions under controlled conditions	Lack whole-organism physiology, systemic immune–metabolic interactions, microbial ecosystem complexity, multicellular crosstalk, and dynamic gut–brain communication; concentration and exposure conditions may not reflect physiological states

Note: The table summarizes the principal contributions and methodological limitations of major human observational and experimental approaches used to investigate the gut–immune–brain axis. Emerging prospective and longitudinal human studies strengthen the evidence for temporal associations between microbiome-related features and cognitive trajectories; however, repeated functional microbiome and metabolomic measurements remain limited, and current observational evidence does not establish causal directionality. Experimental models provide greater mechanistic resolution but have important limitations in translation to human neuroimmune aging. Accordingly, causal inference requires triangulation across longitudinal human cohorts, functional multi-omics, and complementary experimental models [16,19,20,21,23,24,43,51,78,91,107,116,118,131,156,157,158,159,160,161].

**Table 3:** Translational Biomarkers of gut–immune–brain axis dysfunction and neuroimmune resilience.

Biological Level	Biomarker	Biological and Clinical Relevance	Current Evidence and Major Limitations	Potential Application in Precision Medicine
Microbial metabolic signaling	Short-chain fatty acids (SCFAs): acetate, propionate, butyrate	Functional products of microbial fiber fermentation. Butyrate supports colonocyte metabolism, epithelial tight-junction integrity, immune tolerance and microglial homeostasis through receptor-mediated and epigenetic mechanisms. Altered SCFA profiles may indicate disturbed microbial metabolic signaling, although fecal concentrations reflect the balance between production, absorption, utilization, and intestinal transit [31,32,46,51,59].	Human studies support associations between altered microbial metabolite profiles, inflammation and cognitive or mobility decline, but findings vary according to diet, sample type, analytical platform and disease stage. No standardized circulating or fecal cutoff defines neuroimmune dysfunction.	Identification of a metabolite-deficient endotype; selection and monitoring of fiber-rich dietary interventions, precision nutrition, psychobiotics or SCFA-directed postbiotics.
Microbial metabolic signaling	Tryptophan-derived indoles	Microbial indole derivatives influence epithelial integrity, aryl hydrocarbon receptor signaling, immune regulation and host–microbial communication. An altered balance between protective indole pathways and host kynurenine metabolism may accompany inflammatory and neurodegenerative states [52,59,68,69,70,71,172,173].	Metabolomic studies increasingly identify altered host–microbial metabolic networks in aging and AD, but individual indole metabolites remain insufficiently standardized and are strongly influenced by diet, renal function, medication use and sample handling.	Functional metabolic endotyping; distinction between preserved microbial signaling and inflammation-associated diversion of tryptophan toward host kynurenine metabolism; pharmacodynamic monitoring of postbiotic or nutritional interventions.
Microbial metabolic signaling	Primary and secondary bile acids	Reflect combined hepatic and microbial metabolism. Altered primary-to-secondary bile acid profiles may indicate impaired microbial biotransformation and may influence metabolic, immune and neurovascular signaling [28,59,172,173].	Lipidomic and metabolomic cohorts support altered bile-acid profiles and related metabolic pathways in AD and cognitive aging, but results are population- and platform-dependent. Individual bile acids are not disease-specific and are affected by liver function, diet, medication use and enterohepatic circulation.	Definition of a bile-acid dysmetabolism endotype; monitoring of dietary, microbial or metabolic interventions; integration into multi-metabolite panels rather than use as isolated biomarkers.
Intestinal barrier dysfunction	Zonulin-related immunoreactivity	Commonly used as a surrogate indicator of tight-junction regulation and intestinal permeability [207].	Commercial zonulin ELISA assays may detect proteins other than pre-haptoglobin-2, and circulating concentrations correlate poorly with direct intestinal permeability assays. Zonulin should therefore be considered an exploratory rather than a validated standalone biomarker of intestinal barrier dysfunction.	Exploratory component of a multidimensional intestinal barrier panel; interpretation should preferably be combined with functional permeability testing and complementary biomarkers such as I-FABP, LBP, or other validated barrier injury markers.
Microbial translocation/barrier dysfunction	Lipopolysaccharide-binding protein (LBP)	Acute-phase protein that binds bacterial LPS and reflects host exposure to Gram-negative microbial products. It may reflect host exposure to bacterial LPS and associated acute-phase and innate immune responses, but it is not a direct or gut-specific measure of intestinal permeability [50,75,78,79].	Plasma LBP has shown associations with direct permeability measures in human cohorts, but it is an indirect marker and is influenced by hepatic acute-phase responses, obesity, infection and metabolic disease. It does not distinguish gut-derived LPS from other sources.	Identification of an endotoxemia- or translocation-dominant endotype; enrichment of trials targeting barrier integrity; monitoring of diet, postbiotic or anti-inflammatory interventions. Associations with direct permeability measurements have been reported in some human cohorts, but findings are not sufficiently consistent to validate LBP as a direct permeability marker.
Microbial translocation	Circulating LPS/endotoxin activity	Circulating LPS activates TLR4-dependent innate immune signaling, promotes NF-κB activation and inflammasome priming, thereby linking intestinal barrier dysfunction with systemic inflammation [208].	Considerable structural heterogeneity of LPS, differences among bacterial species, supramolecular organization, and limited assay standardization substantially affect LPS detection and biological activity measurements. Consequently, quantification remains technically challenging and inter-study comparability is limited.	Primarily a mechanistic research biomarker; broader clinical implementation requires standardized, biologically validated assays capable of reliably quantifying functionally relevant endotoxin activity.
Systemic inflammaging	Interleukin-6 (IL-6)	A major circulating mediator and biomarker associated with inflammaging, vascular dysfunction, cognitive decline and adverse aging outcomes. Reflects systemic inflammatory burden rather than a gut-specific process [9,102,106,112,114,154].	Prospective and cross-sectional studies support associations between peripheral inflammatory profiles, brain atrophy and cognition, but IL-6 is nonspecific and influenced by infection, obesity, physical activity, comorbidity and medication use.	Identification of an inflammaging-dominant endotype; risk enrichment and pharmacodynamic monitoring in multimarker panels.
Systemic inflammaging	Tumor necrosis factor-α (TNF-α)	Major mediator of chronic innate immune activation, endothelial dysfunction, insulin resistance and neuroimmune signaling [9,102,106,112,114,152].	Associations with cognitive and neurodegenerative outcomes are heterogeneous. Circulating concentrations are often low and variable, and peripheral levels may not directly reflect CNS TNF signaling. Best interpreted with IL-6, CRP, chemokines and metabolic variables.	Characterization of systemic inflammatory phenotypes; mechanistic monitoring rather than disease-specific diagnosis.
Chronic immune activation	Soluble urokinase plasminogen activator receptor (suPAR)	Stable biomarker of chronic immune activation that reflects cumulative inflammatory burden across multiple immune cell types and predicts adverse outcomes in several systemic diseases [209].	Although biologically plausible, prospective evidence does not support baseline circulating suPAR as an independent predictor of cognitive decline or dementia. Current evidence for neurodegeneration-specific prognostic use therefore remains limited and requires further longitudinal validation.	Potential component of a multidimensional systemic inflammation or biological resilience panel rather than a standalone biomarker for cognitive-risk stratification.
Chemokine-mediated inflammation	Monocyte chemoattractant protein-1 (MCP-1/CCL2)	MCP-1/CCL2 promotes CCR2-dependent recruitment and trafficking of monocytes and may reflect signaling between systemic inflammation, the neurovascular unit, and neuroinflammatory responses [81,86,91,116,206].	Altered MCP-1/CCL2 concentrations have been reported in numerous inflammatory, vascular, and neurological conditions, resulting in low disease specificity. Measurements may be influenced by biological compartment, assay platform, renal function, acute inflammatory activity, and systemic comorbidity.	Potential component of an immune-cell recruitment endotype and a multimarker inflammatory panel; primarily exploratory.
Astroglial activation	Glial fibrillary acidic protein (GFAP)	Marker of reactive astrogliosis. Plasma GFAP is associated with amyloid pathology, brain atrophy and subsequent cognitive decline and may rise during preclinical and symptomatic AD. Higher GFAP has also been associated with faster clinical progression in PD cohorts [210].	Among the more analytically and longitudinally supported biomarkers included in this table, but it is not specific to AD and may increase with other neurological injury, age, renal dysfunction and vascular disease. Performance is improved when combined with disease-specific biomarkers.	Early risk stratification, trial enrichment, monitoring of astroglial activation, and integration with p-tau, Aβ, NfL and imaging markers.
Neuroaxonal injury	Neurofilament light chain (NfL)	Sensitive marker of neuroaxonal damage. Higher blood and CSF NfL concentrations are associated with neuroaxonal injury, brain atrophy, and clinical progression across multiple neurological disorders [205].	Strong analytical and prognostic evidence, but limited disease specificity. Concentrations are influenced by age, renal function, vascular disease, acute neurological injury and body composition.	Quantification of neuroaxonal injury burden, prognosis and longitudinal treatment monitoring; particularly useful as part of disease-specific biomarker panels.
Astrocytic/microglial inflammation	YKL-40 (CHI3L1)	Secreted mainly by activated astrocytes and other inflammatory cells. CSF concentrations reflect glial activation and have been associated with AD clinical stage and progression [211].	CSF evidence is more consistent than plasma evidence. YKL-40 is not specific for neurodegeneration and is elevated in systemic inflammatory, hepatic and neoplastic conditions. Recent synthesis indicates association with AD dementia, but additional longitudinal validation is required.	CSF-based characterization of a glial-inflammatory endotype; possible monitoring marker in mechanistic trials, but not ready for standalone clinical diagnosis.
Microglial activation	Soluble triggering receptor expressed on myeloid cells 2 (sTREM2)	Reflects TREM2-related microglial responses, including activation, lipid sensing and phagocytic adaptation. CSF sTREM2 shows stage-dependent changes and may be particularly informative during early symptomatic phases of AD [206].	Predominantly a CSF research biomarker. Associations vary with disease stage, TREM2 genotype and assay method. Its direction of change may represent either protective microglial activation or maladaptive inflammation, complicating interpretation.	Identification of microglial-response endotypes, disease-stage stratification and monitoring of TREM2- or microglia-directed therapies.
Endothelial activation	Intercellular adhesion molecule-1 (ICAM-1)	Reflects endothelial inflammatory activation and leukocyte adhesion. Higher concentrations may indicate systemic vascular injury relevant to BBB dysfunction and cognitive aging [212].	In a 2025 population-based study of dementia-free older adults, higher serum ICAM-1 and a composite endothelial dysfunction score were associated with poorer global cognition, verbal fluency, attention and executive function. However, ICAM-1 remains nonspecific.	Identification of a vascular–endothelial dysfunction endotype; integration with VCAM-1, vascular risk factors, imaging and BBB measures.
Endothelial activation	Vascular cell adhesion molecule-1 (VCAM-1)	Marker of endothelial activation and leukocyte-endothelial interaction that may contribute to vascular inflammation and impaired neurovascular integrity [212].	Human observational data support associations of VCAM-1 with selected cognitive domains, although the magnitude and consistency of these associations vary across populations and are often more informative when VCAM-1 is integrated into composite endothelial dysfunction scores.	Multimarker assessment of vascular and BBB vulnerability; potential enrichment of vascular-protective intervention studies.
Angiogenic/neurovascular signaling	Vascular endothelial growth factor (VEGF)	Regulates angiogenesis, endothelial survival, BBB biology and neurovascular adaptation. Its effects may be context-dependent: compensatory or neuroprotective at some stages but associated with pathological permeability in others [213].	Regional ADNI analyses have associated higher CSF VEGF with more favorable structural and cognitive measures in selected brain regions and disease contexts, although findings do not support a simple uniformly protective interpretation.	Exploratory marker of neurovascular resilience or repair; should be combined with endothelial, imaging and inflammatory markers rather than interpreted directionally in isolation.
Endothelial glycocalyx injury	Syndecan-1, heparan sulfate, hyaluronan and related glycocalyx-shedding products	Used as circulating indicators of glycocalyx shedding or injury, an important regulator of vascular permeability, mechanotransduction and leukocyte adhesion. Potentially relevant to systemic endothelial injury and BBB vulnerability [214].	Strong mechanistic rationale exists in vascular and critical-care research, but direct validation in neuroimmune aging and neurodegeneration remains limited. Concentrations are influenced by acute inflammation, sepsis, diabetes, renal disease and sample timing.	Exploratory definition of a glycocalyx-injury endotype; potentially useful with ICAM-1, VCAM-1, imaging measures and vascular-risk profiling in future longitudinal studies.

Note: The biomarkers summarized above differ substantially in biological maturity and clinical readiness. GFAP and NfL have comparatively strong analytical and longitudinal support, whereas microbial metabolites, barrier and inflammatory markers, endothelial biomarkers, and glycocalyx-shedding products remain primarily as tools for mechanistic investigation and biological endotyping. None of the gut-derived, inflammatory, endothelial, or vascular biomarkers should currently be interpreted as a disease-specific standalone diagnostic test. Their proposed precision applications remain hypothesis-generating and require prospective validation, assay harmonization, predefined cutoffs, characterization of biological and pre-analytical variability, and demonstration of incremental clinical utility. Their greatest translational value may therefore lie in multidimensional panels integrating microbial function, barrier integrity, translocation, systemic inflammation, vascular dysfunction, neuroglial and neuroaxonal injury, clinical variables, and neuroimaging [28,31,32,46,50,51,52,59,75,78,79,80,81,82,83,84,85,86,106,117,128,152,162,172,179,204,205,206,207,208,209,210,211,212,213,214].

**Table 4:** Evidence hierarchy and translational readiness of microbiome-targeted interventions for neuroimmune aging and neurodegenerative disorders.

Intervention	Evidence from Animal Models	Human Observational or Mechanistic Evidence	Randomized Clinical Trials	Systematic Reviews/Meta-analyses	Current Translational Status
Mediterranean-, MIND-, and fiber-rich dietary patterns	Preclinical and mechanistic studies support effects of plant-rich and fiber-rich dietary patterns on microbial composition and function, SCFA production, intestinal barrier homeostasis, systemic inflammation, and selected neurobiological outcomes [31,42,47,51,54,66]	Observational studies associate greater adherence to Mediterranean or MIND dietary patterns with healthier cognitive aging and lower rates of cognitive decline, although residual confounding remains possible and the extent to which these associations are microbiome-mediated is uncertain [42,54,66,219].	Randomized trials have produced mixed findings. PREDIMED cognitive substudies reported benefits in selected cognitive measures, whereas a 3-year MIND diet trial found no significant between-group difference in global cognitive change or brain MRI outcomes compared with a control diet that also included mild caloric restriction [219,220].	Recent systematic and umbrella reviews generally support healthy dietary patterns for brain health, but highlight heterogeneity in interventions, adherence, cognitive tests, and neuroimaging outcomes.	Reasonable as a general health and risk-reduction strategy, particularly Mediterranean-style, plant-rich diets. It should not be presented as an established microbiome-mediated disease-modifying treatment for AD or PD [42,54,66,219,220].
Probiotics/psychobiotics	Extensive animal evidence indicates strain-dependent effects on intestinal permeability, inflammatory signaling, HPA-axis activity, microglial activation, synaptic function, and behavior.	Human studies show changes in microbiota, inflammatory and oxidative-stress markers, gastrointestinal symptoms, and occasionally cognition or mood. Effects are highly strain-, dose-, population-, and endpoint-specific.	Small randomized trials have reported changes in cognition, gastrointestinal symptoms, inflammatory or oxidative-stress markers, and selected motor or nonmotor outcomes; however, effects vary substantially according to strain, formulation, population, treatment duration, and endpoint [143,197,221,222].	Meta-analyses report mixed results, with some suggesting improvement in selected cognitive measures and others finding no significant pooled effect on global cognition. Certainty remains limited by clinical and methodological heterogeneity [221].	Promising adjunctive intervention, but not ready for routine neurodegenerative-disease treatment. Future use requires strain-specific evidence, standardized dosing, validated endpoints, and identification of likely responders.
Postbiotics and defined microbial products	Strong mechanistic and preclinical rationale exists for SCFAs, indoles, secondary bile acids, microbial peptides, cell-wall components, and extracellular vesicles in regulating immunity, barriers, mitochondria, and microglia.	Human evidence is mainly based on metabolomic associations and mechanistic biomarker studies rather than trials of standardized postbiotic products [18,28,31,51,52,59,68,69,70,71,198].	Very limited RCT evidence for formally defined postbiotic preparations in neurodegeneration. Evidence cannot be generalized from probiotics or dietary interventions [195,198,199].	No mature meta-analytic evidence establishing clinical efficacy of postbiotics for AD, PD, or age-related cognitive decline. Reviews emphasize definitional, manufacturing, potency-assay, and standardization gaps [195,198].	Experimental but mechanistically attractive. Appropriate for early-phase, biomarker-rich trials using compositionally defined, potency-characterized, and quality-controlled formulations.
Direct SCFA or SCFA-enhancing supplementation	Considerable animal evidence supports roles of acetate, propionate, and butyrate in intestinal integrity, immune regulation, epigenetic control, and microglial biology. Effects may be context- and dose-dependent.	Human studies link altered SCFA profiles with dysbiosis, inflammation, frailty, PD, and cognitive outcomes, but circulating and fecal measurements are not interchangeable and lack validated therapeutic thresholds [31,44,46,51,59,131,142].	A randomized double-blind study published in 2025 included 72 individuals with PD who received propionate plus butyrate, the prebiotic 2′-fucosyllactose, or their combination for six months. All three active-intervention groups showed clinically relevant motor improvement, accompanied by changes in immune and experimental barrier-related measures. However, the absence of an inert placebo group, retrospective trial registration, and lack of independent replication preclude firm conclusions regarding efficacy [222].	No robust meta-analysis currently establishes direct SCFA supplementation as an effective neurodegenerative therapy [222].	Early clinical signal only. Suitable for confirmatory placebo-controlled trials with pharmacokinetic, metabolomic, barrier, immune, and neurological endpoints.
Vitamin D supplementation as a barrier and immune modifier	Animal studies support effects on epithelial tight junctions, immune regulation, neuroinflammation, and microbiome composition.	Vitamin D deficiency is associated with dysbiosis, inflammatory burden, and cognitive impairment, but observational associations are vulnerable to confounding and reverse causality.	Randomized-trial findings are inconsistent. Large trials conducted predominantly in vitamin-D-replete populations have generally not demonstrated cognitive benefit. In the 24-month VitaMIND trial, supplementation did not improve executive function or other cognitive, functional, or well-being outcomes in 620 adults with mild-to-moderate vitamin D deficiency and early cognitive impairment [201,223].	Meta-analyses report either small overall effects or possible benefits in deficient or vulnerable subgroups, but certainty remains limited and heterogeneity is substantial.	Correct established deficiency according to standard clinical practice, but vitamin D should not be presented as an established microbiome-directed or disease-modifying cognitive therapy [200,201,223].
Fecal microbiota transplantation (FMT)	Strong animal evidence demonstrates transfer of metabolic, inflammatory, behavioral, and sometimes cognitive phenotypes. Young-to-aged FMT has produced improvements in selected aging-related outcomes in rodents.	Human evidence remains limited. Small uncontrolled studies and mechanistic cohorts suggest changes in constipation, microbial composition, and selected neurological symptoms.	Randomized trials of FMT in PD have produced inconsistent findings. One phase 2 study reported improvement in motor outcomes after donor FMT, whereas another randomized clinical trial did not demonstrate superiority on its primary motor endpoint. Studies remain small and differ in donor selection, administration route, bowel preparation, dose, control intervention, and engraftment assessment [224,225].	Systematic synthesis indicates possible improvement in disease burden across microbiome-based interventions, but FMT-specific evidence is too heterogeneous and limited for firm conclusions.	Experimental for neurodegenerative indications. Should be restricted to rigorously regulated clinical trials with donor screening, safety surveillance, engraftment assessment, and long-term follow-up [204,224,225].
Defined microbial consortia/next-generation live biotherapeutics	Strong preclinical rationale supports rationally selected consortia designed to restore specific metabolic functions rather than whole-community transfer [26,55,195].	Human evidence in neurodegeneration is minimal; most development has occurred in gastrointestinal, metabolic, or infectious diseases [195,204].	No convincing phase 2/3 RCT evidence for neuroimmune aging, AD, or PD [195,204].	No relevant clinical meta-analysis.	Preclinical to early translational stage. Designed to offer greater compositional control and potential reproducibility than donor-derived FMT, although comparative clinical safety and efficacy in neurodegenerative disorders remain unproven [195,204].
Polyphenol-rich or metabolite-directed nutritional interventions	Preclinical studies support antioxidant, anti-inflammatory, endothelial, mitochondrial, and microglial effects of selected polyphenols and their microbial metabolites; however, effects vary among compounds and experimental models [42,54,59,66,173].	Human observational and short-term intervention studies show changes in circulating or urinary metabolite profiles and selected inflammatory or vascular markers, but bioavailability and microbial conversion vary markedly between individuals [42,54,59,66,172,196].	Human RCTs often evaluate whole dietary patterns or multicomponent supplements, making the independent microbiome-mediated effect of polyphenols difficult to establish.	Reviews suggest possible cognitive and vascular benefits, but evidence is heterogeneous and insufficient to identify a specific compound, dose, or responder phenotype.	Supportive dietary strategy rather than a validated neurotherapeutic intervention. Future trials should measure microbial conversion products and stratify participants by metabotype [42,54,66,196].
Biomarker-guided and endotype-matched microbiome therapy	Animal studies support the principle that baseline microbial and immune states influence treatment response.	Human cohorts demonstrate major heterogeneity in microbial function, permeability, inflammatory burden, and neuroglial injury, supporting the rationale for endotyping.	Few, if any, trials have prospectively assigned participants to microbiome-targeted interventions using externally validated biomarker-defined endotypes. In the 2025 SCFA/prebiotic PD study, treatment response was associated exploratorily with baseline microbial, immune, transcriptional, and permeability-related features, but these analyses were not based on a prospectively validated treatment-selection algorithm [204,205,217,222].	No meta-analysis demonstrates clinical superiority of biomarker-guided over non-stratified microbiome therapy.	High-priority research strategy, not an established treatment model. Requires validated composite panels, prespecified treatment algorithms, calibration, external validation, and prospective biomarker-enriched trials [162,166,204,205,217,218].
Combination approaches	Preclinical studies suggest that diet, metabolites, probiotics, exercise, and immune modulation may act on complementary components of the gut–immune–brain network.	Human mechanistic evidence supports multidomain interactions, but the contribution of individual components is difficult to isolate.	A small 12-week randomized trial in 46 patients with PD compared combined probiotic and vitamin D supplementation with placebo and reported changes in inflammatory, oxidative-stress, gastrointestinal, anxiety, and disease-severity measures. Because both components were administered together, the study cannot determine their individual contributions and requires independent replication [226].	Current syntheses combine heterogeneous microbiome interventions and suggest possible overall benefit, but do not define an optimal combination.	Promising but hypothesis-generating. Factorial, adaptive, or platform trials are needed to identify additive or synergistic effects and avoid unnecessary multicomponent treatment [204,217,226].

Note: Evidence strength and clinical readiness vary substantially across microbiome-targeted interventions. Dietary patterns and probiotics currently have the largest human evidence base, whereas postbiotics, defined microbial consortia, direct metabolite supplementation, and biomarker-guided approaches remain predominantly mechanistic or early translational. FMT has reached randomized evaluation in Parkinson’s disease, but inconsistent findings and unresolved safety, donor/product, engraftment, and standardization issues limit clinical readiness. None of these strategies is currently established as a microbiome-mediated disease-modifying therapy for neurodegenerative disorders. Overall, mechanistic plausibility exceeds clinical validation. Evidence was synthesized from References [16,19,22,26,28,31,32,42,46,51,52,53,54,55,59,66,75,78,85,91,116,118,124,125,126,131,141,142,143,162,166,172,173,195,196,197,198,199,200,201,204,205,217,218,219,220,221,222,223,224,225,226].

**Table 5:** Evidence hierarchy, translational readiness, and potential clinical positioning of microbiome-targeted interventions.

Intervention	Preclinical Evidence	Human Observational and Mechanistic Evidence	RCTs	Systematic Reviews/Meta-analyses	Translational Readiness	Potential Clinical Positioning/Candidate Population
Mediterranean/MIND and fiber-rich dietary patterns [33,54,66,219,220]	+++	+++	++	++	Moderate as a preventive and general health strategy; insufficient evidence for microbiome-mediated disease modification	Highest current practical applicability as a broadly supportive preventive strategy in older adults, particularly when dietary quality, plant-food intake, or fermentable fiber intake is suboptimal; not a disease-specific treatment
Probiotics/Psychobiotics [143,197,221]	+++	++	++	++	Early clinical; potentially adjunctive, but effects remain strain-, formulation-, dose-, and population-specific	Selected patients as adjunctive therapy, ideally when the administered strain, biological target, and clinical phenotype are well-defined; routine class-wide use is not justified
Postbiotics [18,28,31,51,52,59,68,69,70,71,195,198]	+++	+	–	–	Experimental; strong mechanistic rationale but insufficient neurodegeneration-specific clinical evidence	Future candidate for mechanism-defined or metabolite-deficient endotypes, particularly when standardized delivery of microbial bioactive functions is desirable; currently investigational
Direct SCFA or SCFA-enhancing supplementation [31,32,46,51,59,124,199,222]	+++	++	+	–	Early clinical signal requiring independent placebo-controlled confirmation	Potentially relevant to individuals with evidence of reduced SCFA availability or impaired SCFA-related microbial function; optimal formulation, dose, compartment, and treatment-selection thresholds remain undefined
Vitamin D [200,201,223]	++	++	±	±	Recommended for correction of established deficiency; not established as a microbiome-directed or disease-modifying therapy	Most appropriate in individuals with documented vitamin D deficiency or insufficiency, particularly when barrier, metabolic, or inflammatory vulnerability coexists; not recommended as a universal neuroimmune microbiome therapy
FMT [123,131,141,156,157,195,204,224,225,227,228]	+++	+	++	++	Experimental; randomized and meta-analytic evidence does not currently establish efficacy	Restricted to highly selected research populations within controlled clinical trials; not appropriate for routine neurodegenerative care because of donor-, engraftment-, safety-, procedural-, and regulatory uncertainties
Defined microbial consortia/next-generation live biotherapeutics [26,55,195,204]	++	+	–	–	Preclinical to early translational	Potential future option for functionally characterized dysbiosis or pathway-specific deficits requiring more controlled ecosystem manipulation than conventional probiotics; currently research-stage
Biomarker-guided and endotype-matched therapy [46,53,54,162,166,196,204,205,206,207,208,209,210,211,212,213,214,215,216,217,218,222]	+	++	–	–	Precision-research stage; no validated treatment-selection model	Conceptually most relevant for biologically heterogeneous patients in whom microbial, metabolic, barrier, inflammatory, vascular, or neuroglial endotypes can be defined; currently unsuitable for routine treatment selection
Combination strategies [42,54,72,200,204,226]	++	++	+	–	Promising but hypothesis-generating; component-specific and synergistic effects remain undetermined	Potentially relevant to patients with multidomain dysfunction in whom no single intervention adequately addresses microbial, barrier, metabolic, and inflammatory abnormalities; should be evaluated in mechanism-driven trials

Note: Semi-quantitative grading reflects the relative volume, methodological maturity, and consistency of evidence within each domain and does not represent therapeutic effect size or a formal GRADE assessment. +++ = comparatively extensive or methodologically mature evidence; ++ = supportive but heterogeneous or limited evidence; + = preliminary or early-stage evidence; ± = clinically conflicting evidence; – = no meaningful intervention-specific evidence currently available. Preclinical and mechanistic evidence should not be interpreted as proof of clinical efficacy. The potential clinical positioning/candidate population column provides a hypothesis-driven interpretation based on evidence maturity, safety, mechanistic specificity, and plausible patient selection and should not be interpreted as a validated treatment algorithm or clinical recommendation. Evidence grading and translational interpretation were synthesized from References [18,26,28,31,32,42,46,51,52,53,54,55,59,66,68,69,70,71,72,123,124,131,141,143,156,157,162,166,195,196,197,198,199,200,201,204,205,206,207,208,209,210,211,212,213,214,215,216,217,218,219,220,221,222,223,224,225,226,227,228].

**Table 6:** Translational roadmap for microbiome-targeted interventions within the GIBRA framework.

Translational Domain	Representative Strategies	Primary Biological Target	Current Evidence	Major Translational Barrier
Restoring microbial signaling	Precision nutrition, psychobiotics, and compositionally defined postbiotics [28,31,32,42,52,59,66,71,195,196,197,198]	SCFAs, indoles, bile acids, and microbial extracellular vesicles	Preclinical to early clinical	Interindividual heterogeneity and product characterization, potency assessment, and standardization
Restoring barrier integrity	Fiber-rich dietary strategies, SCFA-directed interventions, and correction of vitamin D deficiency [31,42,47,54,66,75,76,77,78,85,199,200,201]	Intestinal epithelium, tight junctions, endothelial interfaces, and the BBB	Observational and early interventional	Limited direct evidence that microbiome-targeted interventions restore BBB integrity in humans
Modulating immune aging	Anti-inflammatory dietary patterns, metabolite-based interventions, and immunometabolic modulation [9,10,11,30,31,32,47,91,102,105,106,109,110,111,152,202,203]	Inflammaging, trained immunity, immunometabolic dysfunction, and microglial priming	Predominantly mechanistic and preclinical	Pathway specificity, off-target effects, and long-term safety
Precision microbiome medicine	Multidomain biomarker panels, multi-omics integration, and interpretable AI models [46,162,163,164,165,166,170,171,172,185,186,187,188,189,190,191,192,193,204,205,206,207,208,209,210,211,212,213,214,215,216,217,218]	Functional endotyping, risk stratification, and treatment-response prediction	Emerging	Assay reproducibility, computational standardization, interpretability, calibration, and external validation
Clinical implementation	Biomarker-enriched and adaptive trials, longitudinal monitoring, and mechanism-matched treatment allocation [162,166,195,204,205,217,218]	Patient stratification, target engagement, treatment matching, and response monitoring	Early-stage	Standardization, regulation, prospective validation, and demonstration of clinical utility
Ecosystem restoration	FMT and compositionally defined microbial consortia [26,55,123,141,195,204,224,225,227,228]	Whole-community function and restoration of selected microbial metabolic capacities	Experimental for neurodegenerative indications	Donor and product variability, safety, engraftment, ecological stability, manufacturing control, and regulation

Note: Proposed translational roadmap for microbiome-targeted interventions within the Gut–Immune–Brain Resilience Axis (GIBRA), organized according to biological objective, representative strategy, evidence maturity, and major barriers to clinical implementation. The framework emphasizes biomarker-guided, mechanism-based, and endotype-informed approaches rather than universal microbiome-directed interventions and recognizes that the preservation or restoration of neuroimmune resilience may require the coordinated modulation of microbial signaling, barrier integrity, immune homeostasis, and ecosystem function [16,19,26,31,53,54,59,85,91,162,166,186,195,196,197,198,204,205,206,227,228].

## Data Availability

No new data were created or analyzed in this study. Data sharing is not applicable to this article.

## Abbreviations

The following abbreviations are used in this manuscript:

AD	Alzheimer’s disease
AI	Artificial intelligence
BBB	Blood–brain barrier
CD33	Cluster of differentiation 33
CNS	Central nervous system
CR1	Complement receptor 1
DNA	Deoxyribonucleic acid
FMT	Fecal microbiota transplantation
GFAP	Glial fibrillary acidic protein
GIBRA	Gut–Immune–Brain Resilience Axis
GM-CSF	Granulocyte-macrophage colony-stimulating factor
HDAC	Histone deacetylase
HIF-1α	Hypoxia-inducible factor-1 alpha
IDO1	Indoleamine 2,3-dioxygenase 1
IFN-γ	Interferon gamma
IL	Interleukin
IL-1β	Interleukin-1 beta
IL-6	Interleukin-6
IL-17	Interleukin-17
IL-18	Interleukin-18
LBP	Lipopolysaccharide-binding protein
LIME	Local Interpretable Model-Agnostic Explanations
LPS	Lipopolysaccharide
MAMPs	Microbial-associated molecular patterns
NF-κB	Nuclear factor kappa B
NLRP3	NOD-like receptor family pyrin domain-containing 3
NMDA	N-methyl-D-aspartate
NOD	Nucleotide-binding oligomerization domain
PD	Parkinson’s disease
RNA	Ribonucleic acid
SASP	Senescence-associated secretory phenotype
SCFAs	Short-chain fatty acids
scRNA-Seq	Single-cell RNA sequencing
SHAP	SHapley Additive exPlanations
TLR2	Toll-like receptor 2
TLR4	Toll-like receptor 4
TLR5	Toll-like receptor 5
TNF-α	Tumor necrosis factor alpha
TREM2	Triggering receptor expressed on myeloid cells 2
ZO-1	Zonula occludens-1
16S rRNA	16S ribosomal ribonucleic acid
Aβ	Amyloid-beta
α-Synuclein	Alpha-synuclein
γδ T cells	Gamma delta T cells
